# The genome of African manatee *Trichechus senegalensis* reveals secondary adaptation to the aquatic environment

**DOI:** 10.1016/j.isci.2024.110394

**Published:** 2024-06-28

**Authors:** Xin Huang, Guixin Dong, Huizhong Fan, Wenliang Zhou, Guangping Huang, Dengfeng Guan, Delu Zhang, Fuwen Wei

**Affiliations:** 1 Center for Evolution and Conservation Biology, Southern Marine Science and Engineering Guangdong Laboratory (Guangzhou), Guangzhou 511458, China; 2 CAS Key Laboratory of Animal Ecology and Conservation Biology, Institute of Zoology, Chinese Academy of Sciences, Beijing 100101, China; 3 University of Chinese Academy of Sciences, Beijing 100049, China; 4 Guangdong Chimelong Group, Co., Ltd., Guangzhou 511400, China; 5 Jiangxi Provincial Key Laboratory of Conservation Biology, College of Forestry, Jiangxi Agricultural University, Nanchang 330045, China; 6 Chimelong Ocean Kingdom, Zhuhai 519000, China

**Keywords:** Zoology, Molecular biology, Evolutionary biology

## Abstract

Sirenians exhibit unique aquatic adaptations, showcasing both convergent adaptive features shared with cetaceans and unique characteristics such as cold sensitivity and dense bones. Here, we report a chromosome-level genome of the African manatee (*Trichechus senegalensis*) with high continuity, completeness, and accuracy. We found that genes associated with osteopetrosis have undergone positive selection (*CSF1R* and *LRRK1*) or pseudogenized (*FAM111A* and *IGSF23*) in the African manatee, potentially contributing to the dense bone formation. The loss of *KCNK18* may have increased their sensitivity to cold water temperatures. Moreover, we identified convergent evolutionary signatures in 392 genes among fully aquatic mammals, primarily enriched in skin or skeletal system development and circadian rhythm, which contributed to the transition from terrestrial to fully aquatic lifestyles. The African manatee currently possesses a small effective population size and low genome-wide heterozygosity. Overall, our study provides genetic resources for understanding the evolutionary characteristics and conservation efforts of this species.

## Highlights

• *De novo* chromosome-level assembly of the African manatee genome
• Genetic basis for increased bone density and enhanced cold sensitivity
• Convergent evolutionary signatures among fully aquatic mammals
• Currently low genome-wide heterozygosity and a small effective population size

## Graphical abstract

**Figure:**
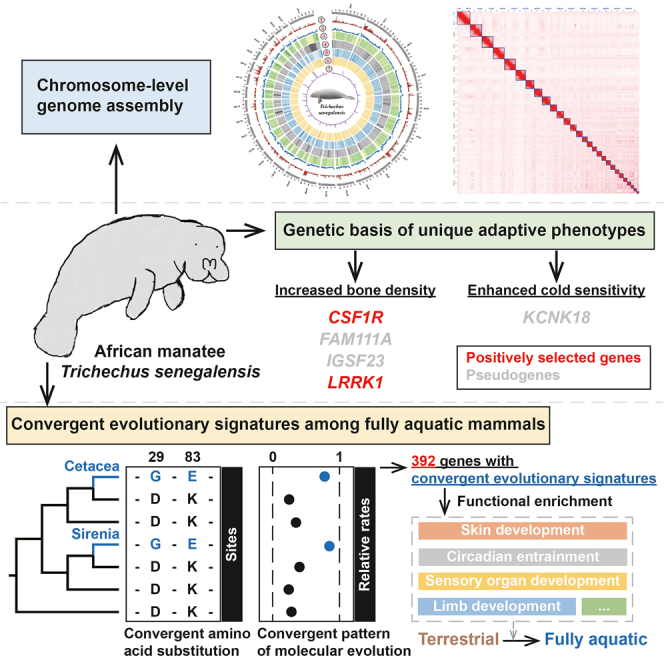

## Introduction

Within the superorder Afrotheria, the Sirenia is an order of placental mammals fully adapted to aquatic environments. Originating in the Paleocene from Tethytheria, a group of hoofed mammals that also gave rise to modern elephants (order Proboscidea), the extant Sirenia include two families, Dugongidae and Trichechidae, and encompassing four species, including dugong (*Dugong dugon*), African manatee (*Trichechus senegalensis*), West Indian manatee (*Trichechus manatus*), and Amazonian manatee (*Trichechus inunguis*).^1^

During the independent transition from land to water, the sirenians share multiple convergent adaptations with cetaceans (order Artiodactyla). For locomotion, all extant sirenians and cetaceans have lost their hind limbs and modified the forelimbs into flippers. For integument, fully aquatic mammals have sparse hair coverage and convergent loss of the sebaceous glands and sweat glands.^2^ These convergent phenotypes are likely to act as hydrodynamic adaptations to facilitate swimming. Besides, the circadian organization of activity and sleep became irregular in fully aquatic mammals. Most obviously, the sirenians and cetaceans convergently adopt a unique unihemispheric slow-wave sleep (USWS). Moreover, the sirenians still retain the bihemispheric slow-wave sleep (BSWS) and rapid eye movement (REM) sleep.^3^ Despite previous studies that have explored the genetic basis behind these convergent adaptations in marine mammals, limited molecular signatures have been identified due to the relatively low quality of the genome assembly and annotation of the West Indian manatee they used to represent the order Sirenia.^4^^,^^5^^,^^6^

The sirenians also evolved unique phenotypes to adapt to their feeding habits and the aquatic environments. They are the only extant marine mammals classified as herbivores.^1^ Associated with their herbivorous lifestyle, manatees exhibit a remarkably low metabolic rate that can drop to as low as 36% of the average for typical placental mammals during food scarcity.^7^ Meanwhile, unlike most fully aquatic cetaceans, the manatees have a thin layer of blubber but thick skin, resulting in poor insulation and sensitivity to temperature changes. It is hard for manatees to thermoregulate in cold water conditions, when the water temperature dips below 20°C; they often migrate to warmer waters. Prolonged exposure to cold water can lead to cold stress syndrome in manatees, potentially resulting in death in severe cases.^8^ Moreover, adult manatees are the extant marine mammals with the strongest bone mass increase.^9^ The dense bones facilitate buoyancy counteraction, allowing them to stay close to the seafloor and the plants on which they prefer to graze without having to expend excessive energy. However, until now, the genetic mechanism underlying these unique adaptations, particularly regarding low-temperature sensitivity and dense bones, has not been explored.

At present, all four extant Sirenia species are listed as “vulnerable (VU)” by the International Union for Conservation of Nature (IUCN). Notably, another Sirenia species, the Steller’s sea cow (*Hydrodamalis gigas*) became extinct in the 18th century.^10^ Climate change and human disturbance are the main factors currently threatening sirenians. Sirenians naturally inhabit rivers, estuaries, marine wetlands, and coastal marine waters, which often overlap with human habitats, potentially exacerbating human-sirenian conflicts. The African manatee likely is the most highly threatened of all the sirenians.^1^ However, until now, there are no population estimates for African manatees based on quantitative information, and there exists a continuous population decline and increased habitat fragmentation.^11^ Hence, genome-wide heterozygosity and demographic history assessments are crucial for conservation efforts.^12^

Herein, based on the newly assembled chromosome-level genome of the African manatee, we explored the possible genetic basis of unique adaptive phenotypes in the African manatee, such as increased bone density and enhanced cold sensitivity. Meanwhile, we identified convergent evolutionary signatures in protein-coding genes among fully aquatic mammals, potentially contributing to their land-to-water transition. Furthermore, population dynamics and genome-wide heterozygosity of three Sirenia species were assessed, emphasizing conservation efforts for African manatees. Taken together, these results will provide useful and valuable genomic resources for future research on the evolution, ecology, and conservation of Sirenia species.

## Results

### Summary of genome assembly and annotation

**Figure 1:**
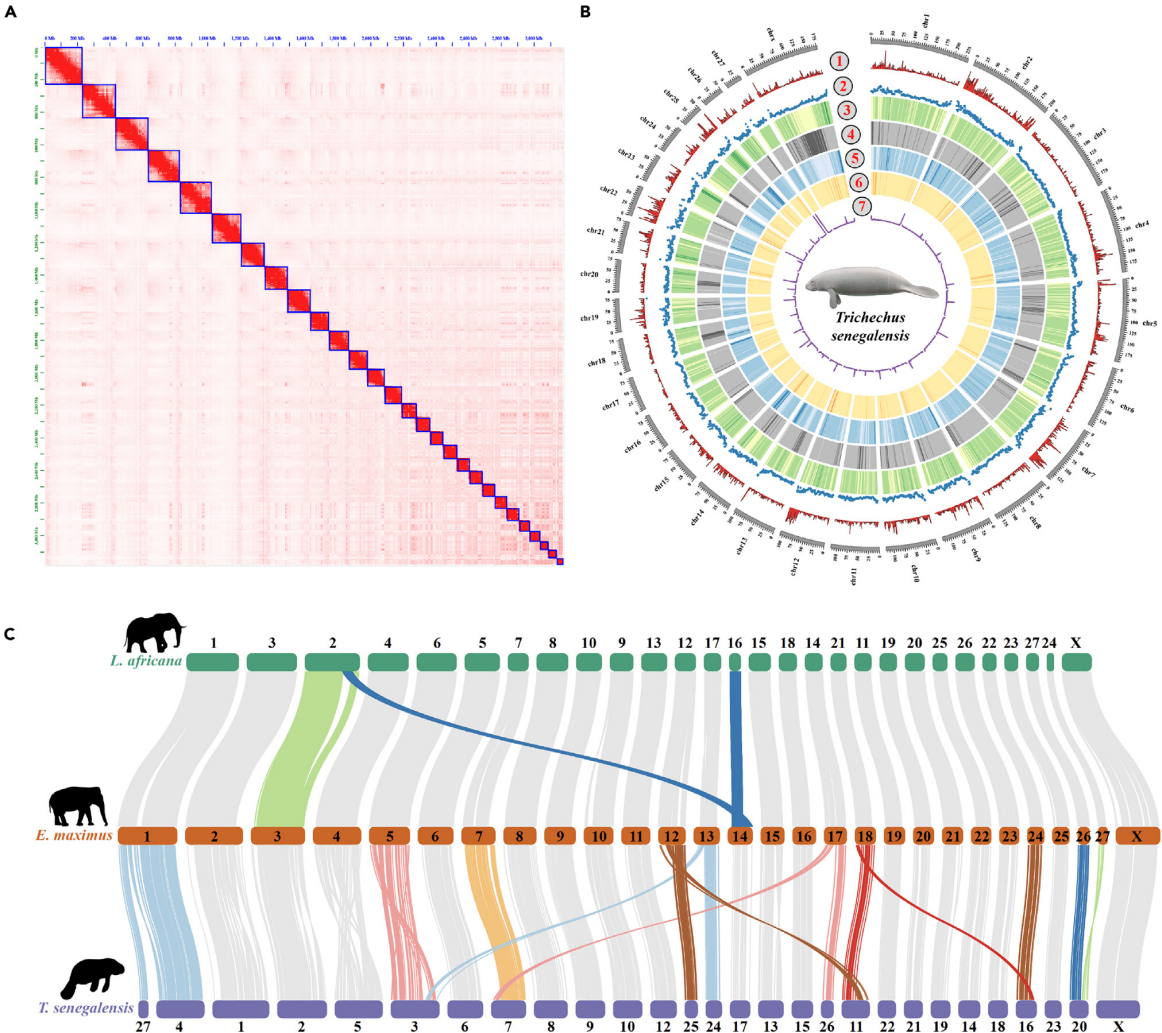
The genomic features and chromosome evolution of the African manatee (A) The Hi-C heatmap of genomic interactions for the African manatee. (B) Circos plot showing the distributions of genomic components with a window of 1 Mb. 1: gene frequency, 2: density of GC content, 3: density of DNA, 4: density of LINEs, 5: density of SINEs, 6: density of LTRs, 7: density of TRs. (C) Chromosome syntenic relationship of the African manatee, Asian elephant, and African elephant; each line represents a syntenic block and the evolutionarily conserved chromosome pairs are shown by gray lines.

Based on the assembled complete mitogenome (16,883 bp; Figure S1), we reconfirmed that the samples we collected were of the African manatee origin (Figure S2). A total of 141.90 Gb HiFi reads, 369.37 Gb Hi-C reads, and 163.10 Gb PE short reads were used for assembling the chromosome-level genome (Table S1). The assembled genome was 3.19 Gb in size, close to the 3.20 Gb estimated by 17 k-mer depth frequency distribution (Figure S3; Table 1). The genome was assembled in only 39 scaffolds, of which 28 represented autosomes and the X chromosome (Figures 1A and 1B). The remaining 11 unplaced scaffolds consisted of only 2.75 Mb combined (0.086% of the total length) (Table S2). The contig N50 value was 118.90 Mb (47 contigs) and the base call accuracy was QV44.90 (0.32 errors per 10,000 bp), indicating the high continuity and accuracy of the assembly. The Hi-C heatmap showing genomic interactions indicates strong agreement between the close interactions and chromosome-length scaffolds (Figure 1A). Mapping of PE and HiFi reads achieved over 99.85% and 100.00% coverage, respectively (Tables 1 and S3). The BUSCO (benchmarking universal single-copy orthologs) analysis showed that 96.3% of the conserved mammalian genes in the mammalian_odb10 dataset were identified as complete (Table S4). Overall, the assessment results showed a high completeness of the assembly.

**Table 1:** Statistics of the assembled African manatee genome

Features	*Trichechus senegalensis*
Genome size (Mb)	3,185.65
Contig N50 (Mb)	118.90
Scaffold N50 (Mb)	136.76
Number of contigs	47
Number of scaffolds	39
Coverage rate for NGS (%)	99.85
Coverage rate for HiFi (%)	100.00
Sequencing depth for NGS (%)	45.86
Sequencing depth for HiFi (%)	44.55
GC content (%)	40.74
Annotated protein-coding genes	20,590
Repeat content (%)	61.69
BUSCO results for assembly	C: 96.3% [S: 95.4%, D: 0.9%], F: 0.9%, M: 2.8%, n: 9226
BUSCO results for annotation	C: 98.0% [S: 97.3%, D: 0.7%], F: 0.1%, M: 1.9%, n: 9226
Base quality (QV)	44.90

Repeat annotation showed that the repetitive elements covered 61.69% of the assembled genome (Figure 1B; Tables S5 and S6). Notably, the SINE (short interspersed nuclear elements) family member AfroSINEs specific for Afrotheria species covered 5.52% of the African manatee genome (Figure S4), which emerged after the split of Afrotheria mammals from the common ancestor shared with Xenarthra.^13^ Among these AfroSINEs, the AFRO_LA accounted for the largest proportion (34.0%) of all AfroSINEs (Figure S4). Meanwhile, the type AFRO_LA has been proven to diverge from AfroSINEs more recently,^13^ which indicated that the type AFRO_LA may have experienced a recent expansion in the African manatee genome.

A total of 20,590 protein-coding genes were predicted based on combined *de novo* gene prediction and homology-based prediction (Table S7), of which, 19,909 genes were functionally annotated by NR and SwissProt databases. Moreover, the basic metrics for the annotated genes of the African manatee are consistent with those of other mammalian species (Figure S5; Table S7). The BUSCO assessment shows that 98.0% of the conserved mammalian genes were annotated to be complete (Table S8).

The collinear relationship among the genome of the African manatee, Asian elephant, and African elephant suggested that there existed relatively conserved karyotypes between these two Proboscidea species and multiple fusion, fission, and translocation events had occurred between the Asia elephant and African manatee (Figure 1C). For instance, there was one chromosome (Chr) fission event from Chr 1 of Asia elephant to Chr 4 and 27 in African manatee, and one fusion event from Chr 26 and 27 to Chr 20.

### Aquatic adaptation in African manatee genome

**Figure 2:**
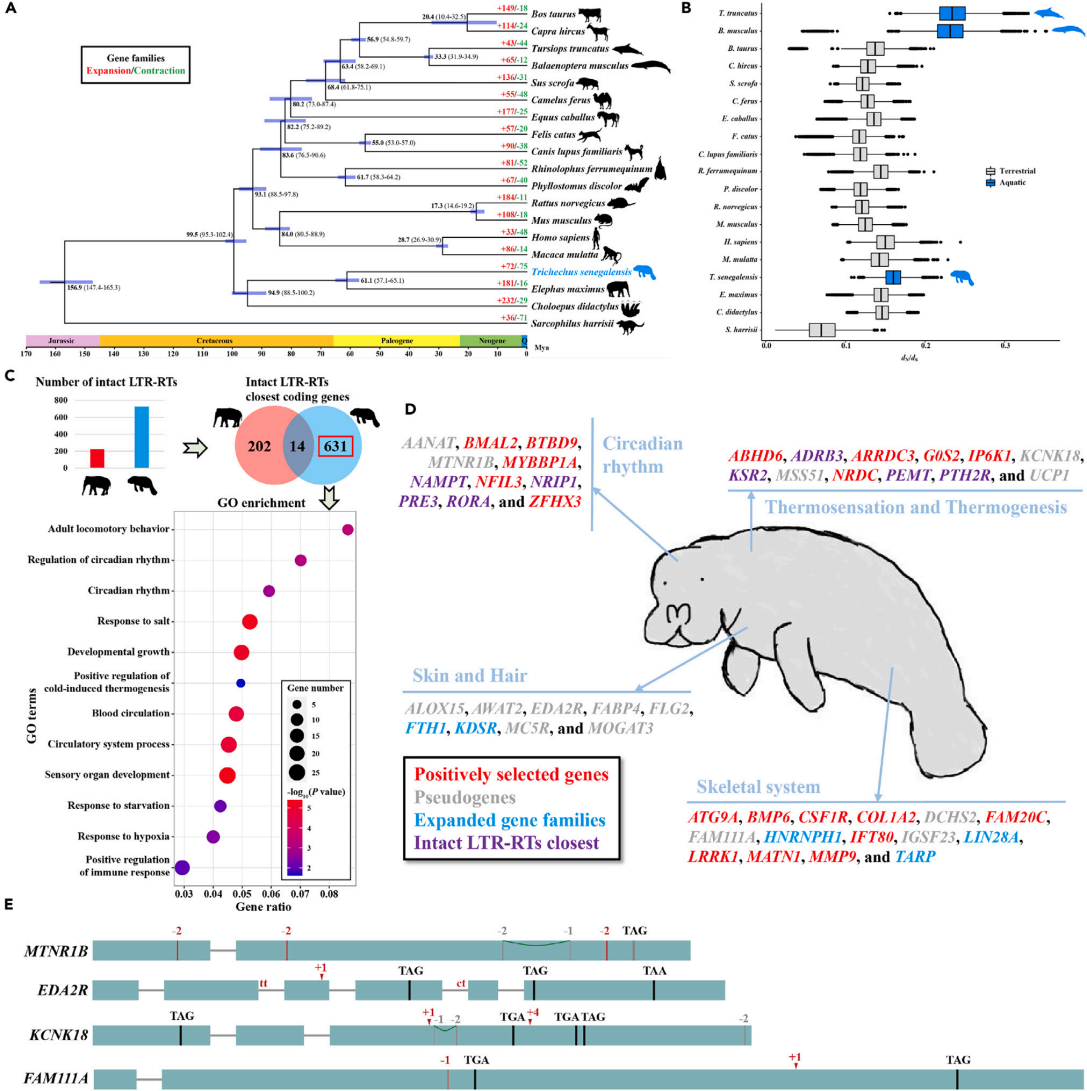
Genomic signatures of aquatic adaptation in African manatee (A) The phylogenetic relationship and estimated divergence time of 19 mammalian species that were used for comparative genomic analysis. The number of significantly expanded (red) and contracted (green) gene families are labeled on each terminal branch. (B) The *d*_N_/*d*_S_ ratio for each species. A total of 8,543 orthologous protein-coding genes were used to calculate the *d*_N_/*d*_S_ ratios under the PAML free-ratio model. (C) The significantly enriched GO terms for African manatee-specific intact LTR-RTs closest genes. The circle size represents the gene number enriched in the corresponding GO terms, and the color of the circles indicates the *p* value of GO terms. The x axis shows the “gene ratio,” or the ratio of enriched genes to all genes in the terms. The histogram and Venn diagram show the number of intact LTR-RTs and their closest genes in the African manatee and Asian elephant genomes, respectively. (D) Genes with signatures of adaptive evolution in African manatee. (E) Exon-intron structure visualization with inactivating mutations that were detected in gene *MTNR1B*, *EDA2R*, *KCNK18*, and *FAM111A* for African manatee. The premature stop codons, frameshifting deletions and insertions, and donor or acceptor splice site mutations are shown in the structure.

We identified 8,543 single-copy orthologous genes within the African manatee and 18 mammalian species. Based on the 4-fold degenerate sites, the divergence times between 19 mammalian species were estimated by the MCMCtree (Figure 2A). As a result, the African manatee and Asian elephant were estimated to diverge at around 61.1 Mya (95% highest posterior density [HPD] = 65.1–57.1). Additionally, the inferred phylogenetic topology for the mammalian species was congruent with previous studies.^14^^,^^15^

The results of gene family alteration showed that the African manatee has 72 significantly expanded and 75 significantly contracted gene families (Figure 2A). For expanded gene families, we found three genes were associated with skeletal development and/or bone density (*HNRNPH1*, *LIN28A*, and *TARP*) and two genes (*FTH1* and *KDSR*) were related to keratosis. Besides, the contracted gene families were mainly enriched in the “sensory perception of smell” (Table S9).

We estimated the *d*_N_/*d*_S_ ratio based on the single-copy orthologous genes to assess the selective pressures acting on the African manatee. We found that the fully aquatic marine mammals owned a higher *d*_N_/*d*_S_ ratio in protein-coding genes when compared with terrestrial species (Figure 2B). 407 positively selected genes (PSGs) were detected in the African manatee after filtering by strict conditions (Table S10). Functional enrichment analyses showed that 78.4% (319/407) PSGs were significantly enriched in 489 Gene Ontology (GO) terms, such as “skeletal system development” (GO:0001501), “circadian rhythm” (GO:0007623), and “regulation of cold-induced thermogenesis” (GO:0120161) (Figure S6; Table S11). In-depth inspection revealed that 25 PSGs of African manatee are related to several aspects of skeletal system development, such as bone development and/or morphogenesis (e.g., *ATG9A*, *BMP6*, *FAM20C*, *IFT80*, *LRRK1*, and *MATN1*) and ossification (e.g., *COL1A2*, *CSF1R*, and *MMP9*). Notably, we found three PSGs were linked to the disease “osteopetrosis” in mice or humans, including *CSF1R*, *LRRK1*, and *MMP9*. We also observed eleven PSGs associated with the regulation of circadian rhythm (e.g., *BMAL2*, *BTBD9*, *MYBBP1A*, *NFIL3*, and *ZFHX3*) (Figure 2D).

Transposable elements play an important role in driving genome evolution by integrating into the genome at a new site.^16^ The African manatee genome has 725 intact LTR-RTs, more than the Asian elephant, which has 223 intact LTR-RTs (Figure 2C; Table S12). We identified 631 specific genes closest to these intact LTR-RTs for the African manatee (Figure 2C). Enrichment analysis showed that these genes were mainly related to “positive regulation of cold-induced thermogenesis” (GO:0120162), “circadian rhythm” (GO:0007623), and “developmental growth” (GO:0048589) (Figures 2C and 2D; Table S13). These intact LTR-RTs may affect the closest gene expressions and contribute to shaping the adaptive phenotypes in the African manatee.

A genome-wide screen revealed 75 protein-coding genes were “lost” and 49 genes were “uncertainly lost” in the African manatee lineage (Table S14), excluding genes belonging to keratin-associated, zinc finger, taste, and olfactory receptor gene families. Among these, we identified ten pseudogenes that were associated with skin or hair diseases (*ALOX15*, *AWAT2*, *EDA2R*, *FABP4*, *FLG2*, *GNLY*, *MC5R*, *MOGAT3*, *NLRP10*, and *SERPINB12*), six (*CD96*, *DCHS2*, *FAM111A*, *IGSF23*, and *LILRB3*) with skeletal development and/or bone density, and two (*AANAT* and *MTNR1B*) with circadian rhythms (Figures 2D, 2E, and S7). For instance, loss-of-function mutations in the gene *IGSF23* were proven to cause osteopetrosis in humans, which is characterized by increased bone mineral density.^17^ The heterozygous mutations in gene *FAM111A* result in impaired skeletal development with small bones, increased bone density, and short stature in humans.^18^ In particular, we found four pseudogenes related to thermosensation and/or thermogenesis (*KCNK18*, *MSS51*, *NTSR2*, and *UCP1*) (Figures 2D, 2E, and S7). For example, the gene *KCNK18* plays a role in many cellular processes such as action potential, muscle contraction, and hormone secretion. A previous study has proven that the *KCNK18*-KO mice exhibited enhanced mechanical and cold sensitivity.^19^ In short, the nociceptive neurons in *KCNK18*-KO mice showed a decreased threshold for activation and the skin nociceptive C-fibres showed an enhanced activation by cold.

### Convergent evolution in fully aquatic mammals

**Figure 3:**
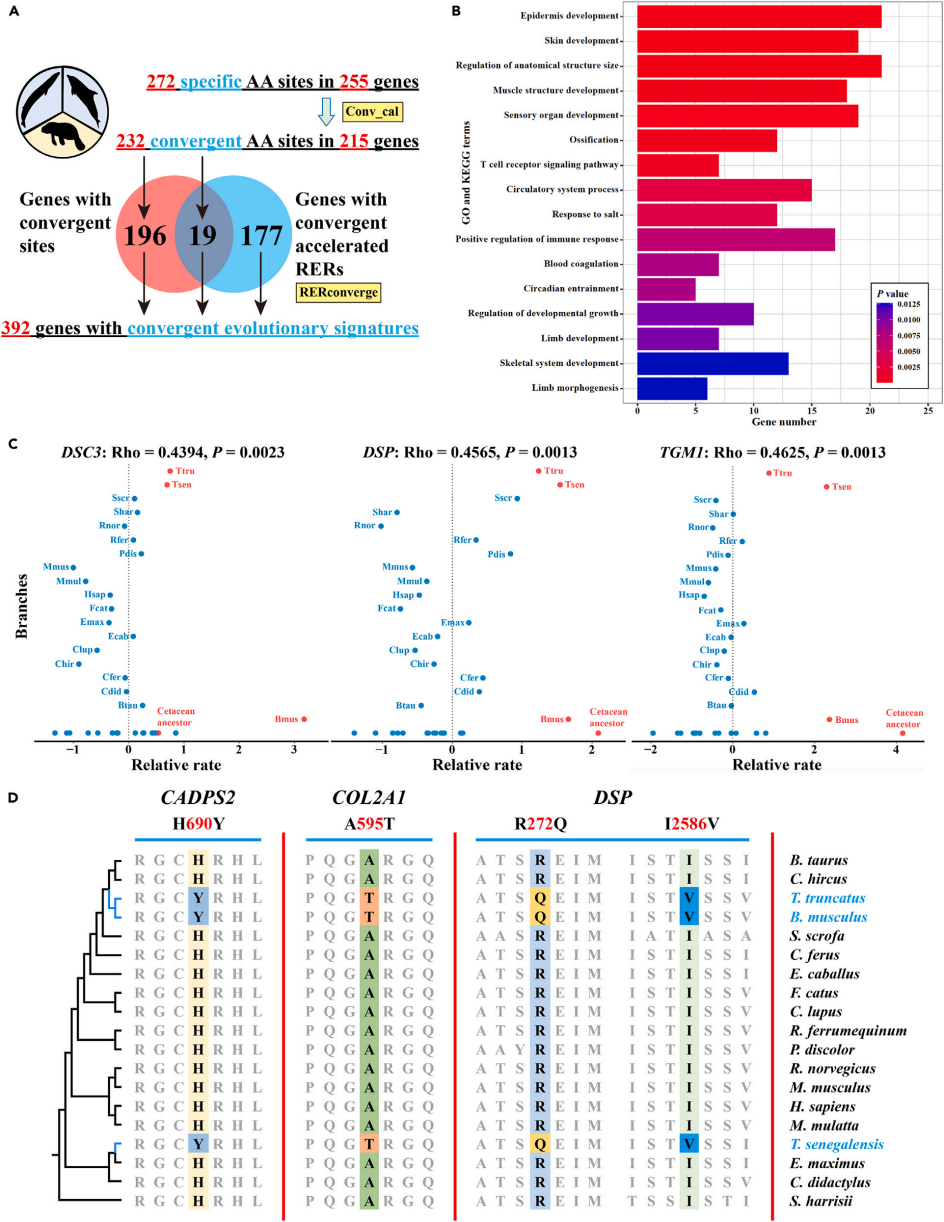
Convergent evolutionary signatures for fully aquatic mammals (A) The pipeline for detecting convergent evolutionary signatures for fully aquatic mammals. (B) The significantly enriched GO and KEGG terms for all genes with convergent evolutionary signatures. The x axis shows the gene number enriched in the corresponding GO and KEGG terms, and the color of the bars indicates the *p* value of GO and KEGG terms. (C) Signatures of convergent evolutionary rate shift in genes *DSC3*, *DSP*, and *TGM1* of fully aquatic mammals. The species names corresponding to the abbreviated names are shown in Table S20. (D) The convergent amino acid substitutions in genes *CADPS2*, *COL2A1*, and *DSP* of fully aquatic mammals. Each of the convergent amino acid substitutions is highlighted in a distinct color. The phylogenetic tree of the 19 mammalian species examined is shown on the left.

We initially found 272 specific amino acid (AA) changes in 255 genes among fully aquatic mammals by FasParser (Figure 3A; Table S15). Among these, 232 AA sites in 215 genes were further identified as convergent substitutions by the conv_cal pipeline (Figure 3A; Table S15). In addition, the RERconverge analyses showed that 196 genes undergoing convergent accelerated relative evolutionary rates (RERs) in fully aquatic mammals (Figure 3A; Table S16). Therefore, a total of 392 genes showed convergent evolutionary signatures, with 19 genes found to have not only convergent AA substitutions but also convergent accelerated RERs (Figure 3A).

Functional enrichment analysis showed that these 392 genes were significantly clustered into 561 GO terms and 37 KEGG pathways (Table S17), such as “skin development” (GO:0043588), “circadian entrainment” (hsa04713), and “limb development” (GO:0060173) (Figure 3B). Further in-depth inspection revealed that 36 genes are associated with “skin or hair disease” (Table S15). For instance, the fully aquatic lineages exhibited convergent accelerated RERs (Figure 3C) and convergent AA substitutions in genes *DSC3*, *DSP*, and *TGM1* (Figures 3D and S8; Table S15). The gene *DSC3* is required for normal desmosome function and maintenance of tissue integrity in the interfollicular epidermis. Adult *DSC3*-null mice showed severe skin lesions, epidermal hyperplasia due to an increase in basal cell proliferation, and complete loss of the hair and epidermis in large sections of the skin.^20^ The gene *DSP* has two convergent AA substitutions (R272Q and I2586V) in fully aquatic mammals (Figure 3D). This gene encodes a desmosomal protein that is critical to cell-cell adhesion; mutations in *DSP* have been proven to cause palmoplantar keratoderma, skin fragility, or woolly hair syndrome.^21^ The gene *TGM1* encodes a catalytic membrane-bound enzyme that functions in the formation of the epidermal cornified cell envelope, which acts as a mechanical barrier to protect against water loss and infectious agents. Mutations in *TGM1* are linked to autosomal recessive congenital ichthyosis (ARCI), a hereditary disorder of cornification, which is mainly characterized by the presence of collodion membrane, alopecia, as well as dry, thickened, and scaly skin.^22^ In addition to the gene *TGM1*, we identified three other genes with convergent evolutionary signatures associated with ARCI, including *ABCA12*, *ALOXE3*, and *PNPLA1* (Tables S15 and S16).^22^ Meanwhile, 36 genes were found to be related to skeletal dysplasia. For example, the gene *COL2A1* has an A595T convergent substitution (Figure 3D), this gene encodes a fibrillar collagen found in cartilage and the vitreous humor of the eye. Heterozygous mutations in the *COL2A1* have been proven to cause a lethal perinatal form of short-limbed dwarfism in humans.^23^ Furthermore, we also found that 9 genes are associated with “circadian rhythm sleep disorder” (Table S15). The gene *CADPS2* has an H690Y convergent substitution (Figure 3D), this gene encodes a protein that is critical for brain-derived neurotrophic factor secretion from neocortical and hippocampal neurons. The *CADPS2*-null mice showed defects in sleep/wake regulation and circadian rhythm.^24^ To sum up, all of these aforementioned genes were associated with the adaptive phenotypes in fully aquatic mammals.

### Population history and genome-wide heterozygosity

**Figure 4:**
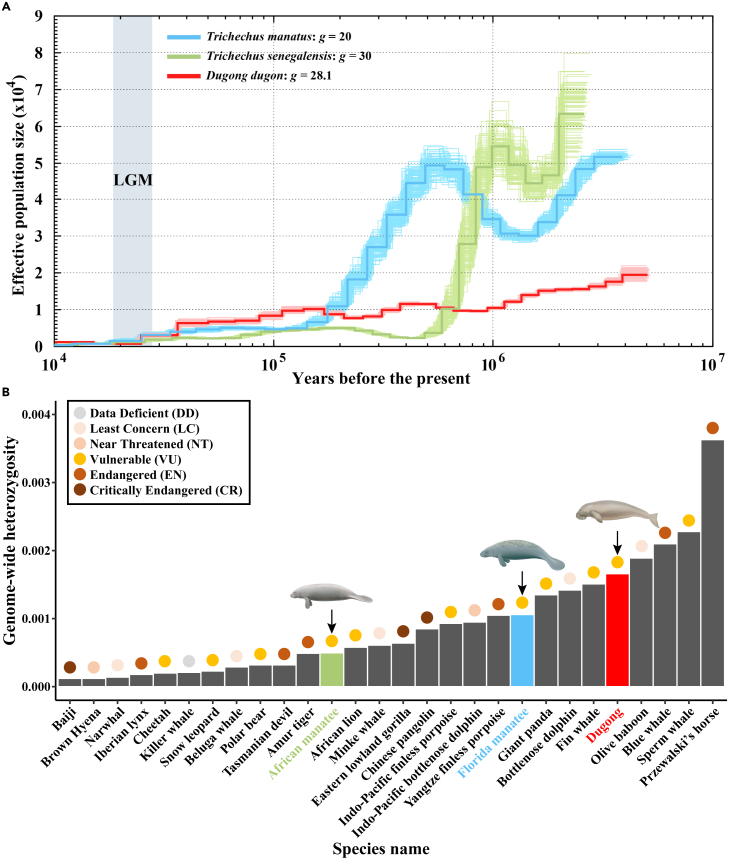
Population history and genome-wide heterozygosity (A) Demographic history inferred by PSMC with 100 bootstraps of the African manatee, Florida manatee, and dugong. (B) The genome-wide heterozygosity of the three Sirenia species and 25 other mammalian species. Circles are colored based on the endangered status listed by the IUCN. The hand-drawn illustrations of the three Sirenia species were obtained from Jefferson et al.^1^

The pairwise sequentially Markovian coalescent (PSMC) model was employed to examine the dynamics in effective population size (*N*_*e*_) of the ancestral populations for three Sirenia species, including the African manatee, Florida manatee (*Trichechus manatus latirostris*), and dugong. The results indicated that all three sirenians showed a population decline during the Last Glacial Maximum (LGM; 26.5 to 19 kya) and all currently exhibit extremely low *N*_*e*_ (Figure 4A).

We identified 1,580,397 high-quality heterozygous single nucleotide polymorphisms (SNPs) in the sequenced African manatee genome (Figure S9), resulting in a frequency of heterozygous sites of 4.97 × 10^−4^, smaller than the Florida manatee (1.06 × 10^−3^) and dugong (1.66 × 10^−3^), which were all listed as “VU” by the IUCN (Figure 4B; Table S18). Compared to other mammals, the African manatee exhibits a relatively low level of genome-wide heterozygosity, specifically, its heterozygosity falls between that of the Amur tiger (*Panthera tigris altaica*, 4.86 × 10^−4^) and the African lion (*Panthera leo*, 5.83 × 10^−4^). It is worth noting that both the Amur tiger and the African lion are classified as “endangered (EN)” and “VU” by the IUCN, respectively.

## Discussion

In this study, we conducted the sequencing, assembly, and annotation of the chromosome-level genome of the African manatee. The genome assembly was produced utilizing circular consensus long-read sequencing technologies, resulting in a high level of continuity and accuracy. Besides, the high-quality African manatee genome assembly permits further discovery of the evolutionary features of the genome and a better understanding of the genetic underpinnings of physiological and morphological secondary aquatic adaptions.

### Genetic basis for secondary aquatic adaptation of the African manatee

#### Skin and hair

The epidermis acts as a stable environmental barrier and performs multiple protective functions for mammals, such as preventing water loss, resisting mechanical stresses, participating in immune responses, and so on.^25^ Layers of the epidermis include the stratum basale, stratum spinosum, stratum granulosum, and stratum corneum, while the Sirenia and Cetacea species lack stratum granulosum.^2^ The skin of manatees is completely cornified, with an extremely thick stratum corneum in which the cells of this layer lack nuclei, unlike the cetaceans, where cells in the stratum corneum retain nuclei and are not fully keratinized. Meanwhile, manatees lack glands throughout the skin, including sebaceous glands (still under debate), sweat glands, and hair follicles, only having blood sinus hair follicles present on the postcranial body.^26^

In our results, we identified convergent evolutionary signatures in gene *TGM1* among fully aquatic mammals. Mutations in *TGM1* were thought to be associated with “autosomal recessive congenital ichthyosis (ARCI),” which was characterized by hyperkeratotic, dry, thickened, scaling skin.^27^ The manatees have finely wrinkled, leathery-looking thick skin that flakes off periodically,^28^ which is phenotypically similar to the ARCI. The convergent evolution of the ARCI-related gene may reflect the adaptation to hydrodynamic movement for fully aquatic mammals, by dramatically increasing the turnover rate of the outermost epidermal cells of the skin.^29^ Moreover, the ARCI-related gene *ALOX12B* and *ALOXE3* owned loss-of-function variants in the Steller’s sea cows, which may be linked to their “the bark of an old oak tree” skin appearance. These two genes are also inactivated in extant cetaceans, but due to the simultaneous loss of the desmosome genes *DSC1* and *DSG4*, the cetaceans have a high shedding rate of stratum corneum, thereby preventing the formation of the ichthyotic leathery skin.^30^ In addition, we also detected convergent evolutionary signatures in other three ARCI-related genes for fully aquatic mammals, including *ABCA12*, *ALOXE3*, and *PNPLA1*. To sum up, the convergent evolution of these ARCI-related genes may play an important role in the adaptive specialization of skin for fully aquatic mammals. Meanwhile, we have discovered that the ichthyosis-related gene *FLG2* was lost in the African manatee, which has also been observed in the dugong.^31^ The *FLG2* gene encodes a filaggrin-like protein that is involved in epithelial homeostasis and is required for proper cornification in the skin. Nonsense homozygous mutations in *FLG2* could induce ichthyosis and generalized peeling skin in humans.^32^ This suggests that the inactivation of the *FLG2* gene may play a significant role in the adaptive modification of the skin barrier as Sirenia species transition from terrestrial to fully aquatic lifestyles.

Particularly, the pelage hairs are greatly reduced in manatees and cetaceans,^26^ and we identified convergent evolutionary signatures in two hairless-related genes *DSC3* and *DSP*. Mutations in these two genes are linked to the loss of hair in humans,^20^^,^^21^ which indicates that convergent evolutionary signatures in *DCS3* and *DSP* may contribute to the reduction of the pelage hair in fully aquatic mammals. In addition, the gene *EDA2R* was pseudogenized in the African manatee genome. The *EDA2R* plays an important role in the maintenance of hair and teeth, and the variations in this gene can cause hypohidrotic ectodermal dysplasia (HED) in humans, which is characterized by abnormal development of the teeth, hair, and sweat glands. A previous study has indicated that HED patients who possess the *EDA2R* polymorphism appeared to have less and thinner hair compared to those who carried the wild-type allele.^33^ That indicates the loss of the *EDA2R* gene may exacerbate the reduction of the pelage in African manatees.

#### Skeletal system

Manatees have evolved dense bones that counteract their buoyancy, allowing them to stay close to the seafloor and the plants on which they graze, without having to expend excess energy. Simultaneously, adult manatees are the extant marine mammals with the strongest bone mass increase.^9^ The pseudogene *IGSF23* and *FAM111A* may play an important role in shaping the dense bones of manatees. It has been proved that the inactivation of the gene *IGSF23* can cause osteopetrosis in humans.^17^ Besides, the heterozygous mutations in gene *FAM111A* result in increased bone density.^18^ Meanwhile, we found three PSGs associated with osteopetrosis, including the *CSF1R*, *LRRK1*, and *MMP9*. The gene *CSF1R* is expressed in osteoclasts, which plays an important role in bone mineralization. The homozygous mutations in *CSF1R* are associated with severe osteopetrosis in rats and mice.^34^ The gene *LRRK1* was thought to play a role in the regulation of bone mass. *LRRK1*-KO mice exhibited severe osteopetrosis, reduced bone resorption, and increased bone mineralization.^35^ To sum up, mutations of these osteopetrosis-related genes may contribute to the increased bone density of the manatees.

Skeletal changes are most extreme in fully aquatic mammals, including hindlimb loss and modification of the front limbs into flippers. Notably, genes with convergent evolutionary signatures in fully aquatic mammals were significantly enriched in limb development-related GO terms. For instance, the gene *COL2A1* has a convergent substitution A595T and showed convergent accelerated RERs. Mutations in the *COL2A1* gene have been found to cause achondrogenesis, which is characterized by short arms and legs or other phenotypes that are related to severe skeletal dysplasia.^23^ We speculated the convergent AA sites in the *COL2A1* may have shaped the convergent limb phenotype of manatees and cetaceans, though functional experiments are required in the future to verify the efficacy.

#### Circadian rhythm

The circadian rhythms dictate alertness or sleepiness, appetite, and body temperature in animals. Most mammals display BSWS and REM sleep, while REM is often minimized for aquatic mammals because the accompanying paralysis can prevent access to air.^36^ In particular, the special USWS has been found in manatees, cetaceans, and eared seals, which allows them to swim and keep one eye open during sleep, besides, monitoring the environment and helps them to awaken rapidly when potential danger is detected.^3^ In our results, we found two melatonin biosynthesis/reception genes *AANAT* and *MTNR1B* were pseudogenized in manatees, which is consistent with the results of Huelsmann et al.^37^ Meanwhile, they identified four genes of melatonin biosynthesis/reception that were lost in cetaceans, including the gene *AANAT*, *ASMT*, *MTNR1A*, and *MTNR1B*, which may have been a precondition to adopt USWS as their exclusive sleep pattern. They also found the gene *ASMT* was inactivated in the West Indian manatee due to the single heterozygous stop codon mutation, but the gene is intact in our genome assembly. Moreover, the African manatee still maintains the intact gene structure of *MTNR1A*. These results mentioned above indicated the manatees may retain parts of the ability for melatonin biosynthesis/reception, which may be the genetic basis for the retention of BSWS and REM sleep in manatees.^3^

The transposable elements play an important role in driving genome evolution by integrating into the genome at a new site.^16^ The African manatee genome displayed a higher number of intact LTR-RTs than those of the relative species Asian elephant, which may reflect recent insertion events. A total of eight genes associated with the circadian rhythm were found to be closest to these intact LRT-RTs. For instance, the gene *RORA* has been shown to aid in the transcriptional regulation of genes involved in circadian rhythm.^38^ The polymorphisms of gene *RORA* were associated with bipolar disorders in humans, which are characterized by major disruptions in circadian rhythms, such as abnormal sleep/wake cycles and alternation in appetite rhythm.^39^ These intact LTR-RTs probably affected gene expressions that contributed to the abnormal circadian rhythm in manatees.

Meanwhile, REM sleep only occupied 1% of 24 h on average for manatees, while cetaceans have lost REM sleep.^40^ Nine genes associated with circadian rhythm sleep disorder exhibited convergent evolutionary signatures among fully aquatic mammals. Among these, *CADPS2*-null mice showed defects in sleep/wake regulation and circadian rhythm; meanwhile, mutations in the *CADPS2* gene induce structural and functional abnormalities of the dorsal raphe nucleus and amygdala, which may lead to REM sleep disorder.^24^ The convergent AA substitution located in the *CADPS2* may play an important role in limiting REM sleep for fully aquatic mammals, although validation requires functional experiments.

#### Cold sensitivity

All four extant Sirenia species are distributed in the tropics and subtropics, have a remarkably low metabolic rate and a thin layer of blubber, and are therefore physiologically intolerant of cold temperatures.^41^ Importantly, manatees exposed to water less than 20°C for several weeks have been shown to die from cold stress syndrome.^8^ Therefore, an enhanced cold sensitivity to the surrounding water temperature is crucial for manatees. Reports of cold-water avoidance behavior further confirm the enhanced cold sensitivity of Sirenia species.^42^^,^^43^ This enhanced cold sensitivity also strongly influences the movement behavior of Sirenia species, resulting in seasonal migrations.^44^ In our results, we found the gene *KCNK18* was pseudogenized in the African manatee. Meanwhile, a recent study confirmed that this gene is also inactivated in the West Indian manatee and dugong.^45^ It has been proved that the nociceptive neurons in *KCNK18*-KO mice showed a decreased threshold for activation and the skin nociceptive C-fibres showed an enhanced activation by cold. Consequently, the cold sensitivity of the *KCNK18*-KO mice was enhanced.^19^ Therefore, the pseudogenization of the *KCNK18* in the African manatee may have enhanced the cold sensitivity so that they can react quickly to fluctuations in water temperature and migrate to warmer waters to reduce damage from acute cold stress.

### Historical declining population and currently low heterozygosity

All four extant Sirenia species are listed as “VU” to extinction by the IUCN, with ongoing population size decline and habitat loss. Our PSMC analysis revealed that all three Sirenia species examined currently possess alarmingly low effective population sizes. It is particularly noteworthy that during the LGM, each of these species has experienced a marked decrease in their *N*_*e*_ and has not yet recovered. The LGM was characterized by plummeting global temperatures and a large drop in sea levels. For instance, the tropical Atlantic Ocean was 5°C cooler than it is today in the LGM.^46^ In particular, the climate change that makes winter months even colder is expected to lead to increased instances of cold stress syndrome in manatees.^47^ The cold stress syndrome can cause death in manatees when they are exposed to cold water temperatures for an extended.^8^ Therefore, we speculate that low water temperatures during the LGM may have contributed to the decline of the sirenian populations. At the same time, the major contemporary threat arises from human disturbance. It is noteworthy that the extinction of the Steller’s sea cow has been regarded as a consequence of human activities, including habitat change and overexploitation.^30^ Furthermore, another human-related threat to the Sirenia species is collisions with watercraft. The Sirenia species have dense bones, rendering them fragile and prone to fractures. Their habitats are highly overlapped with areas of human activity, increasing the vulnerability to life-threatening injuries resulting from boat collisions.

In comparison to other mammals, the African manatee demonstrates a relatively low level of genome-wide heterozygosity. Especially among the three Sirenia species examined, the African manatee has the lowest heterozygosity. The reduced heterozygosity is often associated with reduced reproductive fitness and an increased risk of future extinction. However, until now, the specific number of living individuals and the genetic diversity of African manatees have not been well surveyed. Therefore, considering the currently small effective population size and low genome-wide heterozygosity, as well as the adverse effects of factors such as global temperature fluctuation and human disturbance, more measures are urgently needed to be set up in the future to protect African manatees.

### Conclusion

In this study, we generated a high-quality reference genome for the African manatee. Our findings revealed the potential genetic mechanisms underlying the unique adaptive phenotypes of the African manatee, such as the exceptionally dense bone and sensitivity to cold temperatures. Meanwhile, we found genes with convergent evolutionary signatures among fully aquatic mammals were primarily enriched in skin or skeletal system development, and circadian rhythm, which may contribute to shaping their convergent adaptive phenotypes, correspondingly. Furthermore, the African manatee currently exhibited an extremely small effective population size with the lowest genome-wide heterozygosity among the sirenians examined, which highlights the importance and urgency of the conservation of this EN species. To sum up, our study provides an advantageous basis for future functional experiments, phenotypic and evolutionary biology, and conservation studies on Sirenia species.

### Limitations of the study

Currently, the availability of resources for high-quality genome assembly in Afrotheria species remains limited, potentially obstructing the discovery of more nuanced evolutionary signatures within the Sirenia species. In the future, utilizing recently published high-quality dugong genomes,^45^^,^^48^ we can delve deeper into the genetic basis of distinct adaptive evolutionary traits between manatees and dugongs, such as the tail fluke and skin morphology. Meanwhile, gene function verification experiments *in vivo* and *in vitro* should be further performed to validate the candidate genes we found in this study to be associated with the aquatic adaptive characteristics of the African manatee. Furthermore, it is crucial to gather a broader range of African manatee samples from various regions of their natural habitat to gain a deeper understanding of genetic characteristics for each population, such as genetic diversity, population structures, and inbreeding depression. This knowledge is crucial for establishing conservation units tailored to facilitate the recovery of the population and ultimately safeguard this EN species from further extinction.

## STAR★Methods

### Key resources table

**Table:** 

REAGENT or RESOURCE	SOURCE	IDENTIFIER
**Biological samples**		

Blood samples of the African manatee	Chimelong Ocean Kingdom, Zhuhai, China	N/A

**Deposited data**		

Genome sequencing data	This study	GSA with accession CRA014629
Genome assembly data	This study	GWH with accession GWHERCE00000000

**Software and algorithms**		

fastp (v.0.23.2)	Chen et al.^49^	https://github.com/OpenGene/fastp
MITObim (v.1.9.1)	Hahn et al.^50^	https://github.com/chrishah/MITObim
MITOS web server	Bernt et al.^51^	http://mitos.bioinf.uni-leipzig.de
OGDRAW	Greiner et al.^52^	https://chlorobox.mpimp-golm.mpg.de/OGDraw.html
MACSE (v.2.06)	Ranwez et al.^53^	https://github.com/ranwez/MACSE_V2_PIPELINES
RAxML (v.8.2.12)	Stamatakis^54^	https://github.com/stamatak/standard-RAxML
KmerFreq (v.4.0)	Liu et al.^55^	https://github.com/fanagislab/kmerfreq
GCE (v.1.0.2)	Liu et al.^55^	https://github.com/fanagislab/GCE
Hifiasm (v.0.16.1-r375)	Cheng et al.^56^	https://github.com/chhylp123/hifiasm
purge_dups (v.1.2.5)	Guan et al.^57^	https://github.com/dfguan/purge_dups
Yahs (v.1.2)	Zhou et al.^58^	https://github.com/c-zhou/yahs
Juicebox (v.2.15)	Durand et al.^59^	https://github.com/aidenlab/Juicebox
Tandem Repeat Finder (v.4.10.0)	Benson^60^	https://github.com/Benson-Genomics-Lab/TRF
RepeatModeler (v.2.0.3)	Flynn et al.^61^	https://github.com/Dfam-consortium/RepeatModeler
RepeatMasker (v.4.1.3)	Chen^62^	https://github.com/rmhubley/RepeatMasker
RepeatProteinMask (v.4.1.3)	Chen^62^	https://github.com/rmhubley/RepeatMasker
RepBase database (v.20181026)	Jurka et al.^63^	https://www.girinst.org/server/RepBase
Augustus (v.3.2.1)	Stanke et al.^64^	https://github.com/nextgenusfs/augustus
GeMoMa (v.1.9)	Keilwagen et al.^65^	http://www.jstacs.de/index.php/GeMoMa
EvidenceModeler (v.1.1.1)	Haas et al.^66^	https://github.com/EVidenceModeler
Diamond (v.2.0.14.152)	Buchfink et al.^67^	https://github.com/bbuchfink/diamond
BUSCO (v.5.4.3)	Simão et al.^68^	https://busco.ezlab.org
BWA (v.0.7.17-r1188)	Li and Durbin^69^	https://github.com/lh3/bwa
SAMtools (v.1.16)	Li et al.^70^	https://github.com/samtools/samtools
Merqury (v.1.1)	Rhie et al.^71^	https://github.com/marbl/merqury
CIRCOS (v.0.69.9)	Krzywinski et al.^72^	http://circos.ca/software/download/circos
LASTZ (v.1.02.00)	Harris^73^	https://github.com/lastz/lastz
RectChr (v.1.37)	N/A	https://github.com/BGI-shenzhen/RectChr
OrthoFinder (v.2.5.4)	Emms and Kelly^74^	https://github.com/davidemms/OrthoFinde
Gblocks (v.0.91b)	Castresana^75^	http://molevol.cmima.csic.es/castresana/Gblocks
PAML package (v.4.9e)	Yang^76^	http://abacus.gene.ucl.ac.uk/software/paml.html
TimeTree database	Kumar et al.^77^	http://timetree.org
Café v4.2	De Bie et al.^78^	Éhttps://github.com/hahnlab/CAFÉ
LTR_FINDER (v.1.07)	Xu and Wang^79^	https://github.com/xzhub/LTR_Finder
LTRharvest (v.1.6.2)	Ellinghaus et al.^80^	https://github.com/genometools/genometools
LTR_retriever (v.2.9.0)	Ou and Jiang^81^	https://github.com/oushujun/LTR_retriever
TOGA (v.1.0)	Kirilenko et al.^82^	https://github.com/hillerlab/TOGA
FasParser (v.2.13.0)	Sun^83^	https://github.com/Sun-Yanbo/FasParser
conv_cal pipeline (v.0.3)	Zou et al.^84^	https://github.com/ztzou/conv_cal
RERconverge (v.0.3.0)	Kowalczyk et al.^85^	https://github.com/nclark-lab/RERconverge
Metascape	Zhou et al.^86^	https://metascape.org/gp/index.html
Online Mendelian Inheritance in Man (OMIM)	Hamosh et al.^87^	https://www.omim.org
PSMC (v.0.6.5-r67)	Li and Durbin^88^	https://github.com/lh3/psmc
BCFtools (v.1.16)	Danecek et al.^89^	https://github.com/samtools/bcftools
Genome Analysis Toolkit (GATK) (v.4.2.6.1)	McKenna et al.^90^	https://github.com/broadinstitute/gatk

### Resource availability

#### Lead contact

Further information and requests can be directed to Prof. Fuwen Wei (weifw@ioz.ac.cn).

#### Materials availability

The study did not generate new unique reagents.

#### Data and code availability

• The genome assembly reported in this study has been deposited in the Genome Warehouse at the National Genomics Data Center, Beijing Institute of Genomics, Chinese Academy of Sciences/China National Center for Bioinformation (GWH: GWHERCE00000000). The raw sequencing data for the PacBio HiFi reads, NGS paired-end reads, and Hi-C linked reads that used for genome assembly have been deposited in the Genome Sequence Archive at the National Genomics Data Center, Beijing Institute of Genomics, Chinese Academy of Sciences/China National Center for Bioinformation (GSA: CRA014629). All datasets are publicly available as of the date of publication.
• This paper does not report original code.
• Any additional information required to reanalyze the data reported in this work paper is available from the lead contact upon request.

### Method details

#### Genome sequencing

Blood samples were collected from a captive female manatee named “Xixi” at Chimelong Ocean Kingdom, Zhuhai, China. Animal care and experiments were conducted according to the guidelines established by the Regulations for the Administration of Affairs Concerning Experimental Animals (Ministry of Science and Technology, China, 2017). All procedures were also conducted following the approval of the Animal Experiment Ethics Committee in the Institution of Zoology, Chinese Academy of Sciences, China.

Genomic DNA extraction was performed using the QIAGEN Genomic kit (Cat#13343, Qiagen) for long-read and short-read sequencing. For PacBio HiFi sequencing, SMRTbell target size libraries were constructed according to PacBio’s standard protocol (Pacific Biosciences, CA, USA) using 15 kb preparation solutions, and sequencing was performed on a PacBio Sequel II instrument with Sequencing Primer V2 and Sequel II Binding Kit 2.0 in GrandOmics. The circular consensus analysis was performed in SMRT Link v9.0 under default settings. The paired-end (PE) library with a 350 bp insert size was constructed following the manufacturer’s instructions, and sequencing was performed on the DNBSEQ-T7 platform with a strategy of 2 × 150 bp. To construct the Hi-C library, the blood samples were first performed cross-linked with formaldehyde. The restriction enzyme *DnpⅡ* was then added to digest the chromatin into units, marked by incubating with biotin-14-dCTP and ligated the units by biotinylation. The ligated DNA was finally sheared into 300–600 bp fragments and was also sequenced on the DNBSEQ-T7 platform with a strategy of 2 × 150 bp.

#### Mitogenome assembly and annotation

Before the mitogenome assembly, adapter sequences and low-quality bases from PE short reads were trimmed by fastp (v.0.23.2).^49^ Based on the clean PE reads, the mitochondrial genome of the sampled manatee was assembled by MITObim (v.1.9.1),^50^ using the West Indian manatee (*T. manatus*) mitogenome (GenBank ID: NC_010302.1) as a reference. The mitogenome annotation was using the MITOS web server,^51^ and was visualized using OGDRAW.^52^ Thirteen mitochondrial protein-coding genes of the sampled manatee and its related species (Table S19) were selected to reconstruct the phylogeny. The multiple sequence alignment (MSA) was performed with MACSE (v.2.06).^53^ Then, these genes were concatenated and used to infer the maximum likelihood (ML) phylogenetic tree by RAxML (v.8.2.12)^54^ with 1,000 bootstraps. As a result, the specimen used for genome sequencing was clustered together with the African manatee with high support (bootstrap value = 100).

#### Nuclear genome assembly, annotation, and assessment

Prior to the genome assembly, based on clean PE reads, we estimate the genome size of the African manatee by KmerFreq (v.4.0)^55^ and GCE (v.1.0.2),^55^ utilizing a k-mer frequency spectrum (k = 17). To produce a chromosome-level African manatee genome assembly, the PacBio HiFi reads were firstly assembled into contigs using Hifiasm (v.0.16.1-r375)^56^ with default parameters, and the purge_dups (v.1.2.5)^57^ was used to remove haplotypic duplication and increase continuity. The Hi-C reads were then used to anchor contigs onto chromosomes with the Yahs (v.1.2).^58^ Finally, the Juicebox (v.2.15)^59^ was used manually to correct assembly errors and adjust the position of the scaffolds based on the Hi-C heatmaps.

For genome annotation, we initially identified repetitive sequences using different software programs. In brief, tandem repeats were predicted by Tandem Repeat Finder (v.4.10.0)^60^ with the parameter “2 7 7 80 10 50 500 -f -d -h -r.” RepeatModeler (v.2.0.3)^61^ was used to build the ab initio repeat library. RepeatMasker (v.4.1.3)^62^ and RepeatProteinMask (v.4.1.3)^62^ were then applied against the ab initio repeat library and RepBase database (v.20181026)^63^ library separately to search for homologous and novel repeats. Protein-coding genes were predicted in the repeats-masked genome by integrating *de novo* prediction and homology-based prediction. First, Augustus (v.3.2.1)^64^ was used to generate *de novo* prediction with internal gene models. Second, the homology-based prediction was performed with GeMoMa (v.1.9).^65^ Finally, all results were integrated into the final gene set using EvidenceModeler (v.1.1.1).^66^ The gene functions were annotated by the SwissProt and NR databases with Diamond (v.2.0.14.152).^67^

Genome assembly and genome annotation completeness were assessed using BUSCO (v.5.4.3)^68^ with mammalian_odb10 gene sets. HiFi reads and PE clean short reads were mapped to the assembled genome using BWA (v.0.7.17-r1188),^69^ respectively. The mapping ratio and genome coverage were calculated with SAMtools (v.1.16).^70^ Base accuracy (QV) was measured using k = 21 with Merqury (v.1.1).^71^ The CIRCOS (v.0.69.9)^72^ was used to visualize the genome features. Additionally, we analyzed the collinearity of the chromosome-level genome assembly of the African manatee and its related species Asian elephant (*Elephas maximus*) and African elephant (*Loxodonta africana*) using LASTZ (v.1.02.00),^73^ and we visualized the collinearity region and detected the chromosome fusion and fission events using RectChr (v.1.37) (https://github.com/BGI-shenzhen/RectChr).

#### Phylogeny reconstruction and divergence time estimation

To construct the gene dataset for further comparative genomic analysis, the one-to-one orthologous gene clusters were identified from the protein-coding sequences of the African manatee and the other 18 mammalian species (*Bos taurus*, *Capra hircus*, *Tursiops truncatus*, *Balaenoptera musculus*, *Sus scrofa*, *Camelus ferus*, *Equus caballus*, *Felis catus*, *Canis lupus familiaris*, *Rhinolophus ferrumequinum*, *Phyllostomus discolor*, *Rattus norvegicus*, *Mus musculus*, *Homo sapiens*, *Macaca mulatta*, *T. senegalensis*, *E. maximus*, *Choloepus didactylus*, and *Sarcophilus harrisii*) (Table S20) using OrthoFinder (v.2.5.4) pipeline,^74^ which applied an all-against-all BLASTP algorithm. MSAs were performed by MACSE at the codon level. Poorly aligned regions with gaps and nonhomologous fragments were removed using Gblocks (v.0.91b)^75^ with strict parameters (“−t = c, −b5 = n”). For phylogenetic tree construction, all MSAs were concatenated to one supergene. 4-fold degenerate synonymous sites from the supergene were subsequently extracted and used for constructing an ML phylogenetic tree by RAxML with 1,000 bootstraps, under the GTRGAMMA model. Divergence time was estimated using MCMCtree from the PAML package (v.4.9e),^76^ which combines with a molecular clock model. Several fossil-calibrated time points (Table S21) were obtained from the TimeTree database.^77^

#### Expanded and contracted gene families

Gene family expansion or contraction analyses were performed by Café (v4.2)^78^ based on the results from OrthoFinder pipeline. Only gene families with a *p*-value <0.01 were considered as having undergone significant change.

#### Positively selected genes

Based on MSAs identified among 19 mammalian species, the ratio of nonsynonymous substitutions to synonymous substitutions (*d*_N_/*d*_S_; *ω*) for each terminal branch was estimated using the free-ratio model of CODMEL in the PAML package. The branch-site model was implemented to test for PSGs with the African manatee set as the foreground branch. In brief, model A (ma; alternative hypothesis) allows several particular sites on the foreground branch to be under positive selection (*ω* > 1), whereas the null model A (ma0; null hypothesis) assumes that sites may evolve either neutrally (*ω* = 1) or under purifying selection (*ω* < 1). A likelihood ratio test (LRT) was then used to test whether model A was significant as compared with null model A; the *p*-value generated from LRT was corrected by FDR. Besides, the potential positively selected sites (PSSs) were determined using Bayes empirical Bayes posterior probabilities of >0.5, following McGowen et al.^91^ Finally, to reduce potential false positive errors, PSGs with a median interval between PSSs ≤10 amino acids were removed.^92^

#### LTR-RTs analysis

To identify the intact LTR-RTs (long terminal repeat retrotransposons), LTR_FINDER (v.1.07)^79^ and LTRharvest (v.1.6.2)^80^ were first used for initial scanning, and the LTR_retriever (v.2.9.0)^81^ was then used to filter out false positive results. Only the elements that passed the filtering step were regarded as intact LTR-RTs. We performed the pipeline for the genome of the African manatee and Asian elephant. Finally, functional enrichment analysis was performed with the intact LTR-RTs closest protein-coding genes for the African manatee, after filtering genes that overlapped with Asian elephants.

#### Pseudogene identification

The TOGA (v.1.0)^82^ pipeline was utilized to identify pseudogenes in the African manatee, using the human T2T genome (GenBank assembly accession: GCA_009914755.4) as a reference. Considering all the various transcripts of each single gene, the TOGA scanned each gene for the presence of inactivating mutations (including frameshifting mutations, premature stop codons, splice site disrupting mutations, and deletions of entire coding exons). It used the precedence order “intact (I), partially intact (PI), uncertain loss, lost (L), missing (M)” to classify the genes. Finally, for genes that were classified as “UL” or “L,” we manually checked the authenticity of the inactivating mutations by our clean PE reads.

#### Convergent evolution analysis

##### Convergent amino acid sites

We identified the convergent evolutionary AA sites in cetaceans and the African manatee, based on MSAs identified among 19 mammalian species. For cetaceans, we selected the blue whale (*B. musculus*) and the bottlenose dolphin (*T. truncatus*) as the representatives of the parvorder Mysticeti and Odontoceti, respectively. First, the FasParser (v.2.13.0)^83^ was used to screen out the specific AA site changes of the three aquatic mammals (foreground group) compared with terrestrial mammals (background group). We strictly required AA sites to be identical within both groups and to differ between groups, except for the marsupial species Tasmanian Devil (*S. harrisii*), which we allowed to be different from both the foreground and background groups due to the large genetic distance from placental species. Subsequently, we further detected convergent signals by testing whether the observed number of convergent substitutions in the candidate genes identified in the first step among three marine mammals significantly exceeds the expected (neutral) number. In brief, we first inferred the ancestral amino acids at all internal nodes across the phylogeny by conv_cal pipeline (v.0.3)^84^ and calculated the observed and expected numbers of convergent substitutions in candidate genes. The AAML program in PAML 4.9e that was applied in this pipeline was performed under the parameter: the *Empirical + F* model together with the *JTT-f*_*gene*_ matrix and a discrete gamma model with four rate categories. Finally, we used the Poisson cumulative-distribution test to assess the significance of observed and expected numbers in the target gene to filter out noise resulting from random AA substitutions. Finally, genes with a *p*-value <0.05 were considered to have undergone convergent evolution. Otherwise, we defined these filtered sites as marine mammal-specific AA mutations.

##### Convergent accelerated genes

The RERconverge (v.0.3.0)^85^ method was performed to detect the correlation between the evolutionary rates of genes and the evolution of convergent traits across phylogeny. We first used the AAML program in the PAML package to estimate the branch lengths at all internal and terminal nodes. Then, we used the “readTrees” function to estimate the average branch lengths across all genes. The RERs were calculated using the “getAllResiduals” function. Then, we set the fully aquatic mammal-related lineages (including the branch leads to African manatee, bottlenose dolphin, blue whale, and the recently common ancestor of the cetaceans) as targets and performed the “correlateWithBinaryPhenotype” function to test for the significant association between RERs and convergent traits across all branches. Finally, the genes with a Rho >0 and a *p*-value <0.05 are considered to exhibit convergent accelerated RERs.

#### Functional enrichment analysis

KEGG and GO enrichment analyses were performed with Metascape.^86^ Furthermore, we used literature searches and Online Mendelian Inheritance in Man (OMIM)^87^ databases to explore the potential biological functions of each candidate gene associated with the adaptive evolutionary trait of the African manatee.

#### Demographic history and genome-wide heterozygosity inference

The demographic history of the sirenians was inferred by the PSMC (v.0.6.5-r67) method,^88^ including African manatee, Florida manatee, and dugong (Table S22). The BWA was first used to align clean PE reads to their respective reference genome. Then, the consensus sequences were obtained using SAMtools and BCFtools (v.1.16).^89^ PSMC analysis was performed with 100 bootstrap replicates using the parameters: “-N25 -t15 -r5 -p 4 + 25 ∗ 2 + 4 + 6.” The estimated generation time (*g*) of these species was obtained from Pacifici et al.^93^ and the neutral mutation rate (*μ*) was set as 2.5 × 10^−8^, which was calculated as the methods mentioned by Yang et al.^94^ SNPs were then identified with the HaplotypeCaller module in the Genome Analysis Toolkit (GATK) (v.4.2.6.1),^90^ based on the generated short-read pile-up above. Genome-wide heterozygosity was calculated using the formula: the ratio of the number of heterozygous sites to the total number of sites. Meanwhile, the sites with less than 10× coverage were filtered.

### Quantification and statistical analysis

Quantification and statistical analysis used in the genome assembly and comparative genome analysis can be found in the method details.

## Supplemental information

Document S1. Figures S1–S9; Tables S1–S8 and S18–S22

Table S9. GO and KEGG enrichment of expanded and contracted gene families in African manatee, related to Figure 2

Table S10. The 407 positively selected genes identified in the African manatee genome, related to Figure 2

Table S11. GO and KEGG enrichment of positively selected genes in the African manatee genome, related to Figure 2

Table S12. Annotations of intact LTR-RTs in African manatee and Asian elephant genome, related to Figure 2

Table S13. GO and KEGG enrichment for African manatee-specific intact LTR-RTs closest genes, related to Figure 2

Table S14. The 75 lost protein-coding genes and 49 uncertainly lost genes in the African manatee lineage, related to Figure 2

Table S15. The convergent evolutionary or specific amino acid substitutions of fully aquatic mammals, related to Figure 3

Table S16. Genes with convergently accelerated relative evolutionary rates in fully aquatic mammals, related to Figure 3

Table S17. GO and KEGG terms for all genes with convergent evolutionary signatures, related to Figure 3

## References

[1] Jefferson T.A., Webber M.A., Pitman R.L. (2011).

[2] Springer M.S., Guerrero-Juarez C.F., Huelsmann M., Collin M.A., Danil K., McGowen M.R., Oh J.W., Ramos R., Hiller M., Plikus M.V., Gatesy J. (2021). Genomic and anatomical comparisons of skin support independent adaptation to life in water by cetaceans and hippos. Curr. Biol..

[3] Rattenborg N.C., Amlaner C.J., Lima S.L. (2000). Behavioral, neurophysiological and evolutionary perspectives on unihemispheric sleep. Neurosci. Biobehav. Rev..

[4] Foote A.D., Liu Y., Thomas G.W., Vinař T., Alföldi J., Deng J., Dugan S., van Elk C.E., Hunter M.E., Joshi V. (2015). Convergent evolution of the genomes of marine mammals. Nat. Genet..

[5] Hu Y., Wang X., Xu Y., Yang H., Tong Z., Tian R., Xu S., Yu L., Guo Y., Shi P. (2023). Molecular mechanisms of adaptive evolution in wild animals and plants. Sci. China Life Sci..

[6] Yuan Y., Zhang Y., Zhang P., Liu C., Wang J., Gao H., Hoelzel A.R., Seim I., Lv M., Lin M. (2021). Comparative genomics provides insights into the aquatic adaptations of mammals. Proc. Natl. Acad. Sci. USA.

[7] Best R.C. (1983). Apparent dry-season fasting in Amazonian manatees (Mammalia: Sirenia). Biotropica.

[8] Hardy S.K., Deutsch C.J., Cross T.A., de Wit M., Hostetler J.A. (2019). Cold-related Florida manatee mortality in relation to air and water temperatures. PLoS One.

[9] Bianucci G., Lambert O., Urbina M., Merella M., Collareta A., Bennion R., Salas-Gismondi R., Benites-Palomino A., Post K., de Muizon C. (2023). A heavyweight early whale pushes the boundaries of vertebrate morphology. Nature.

[10] Sharko F.S., Boulygina E.S., Tsygankova S.V., Slobodova N.V., Alekseev D.A., Krasivskaya A.A., Rastorguev S.M., Tikhonov A.N., Nedoluzhko A.V. (2021). Steller’s sea cow genome suggests this species began going extinct before the arrival of Paleolithic humans. Nat. Commun..

[11] Diagne L.W.K. (2014).

[12] Wei F., Huang G., Guan D., Fan H., Zhou W., Wang D., Hu Y. (2022). Digital Noah’s Ark: last chance to save the endangered species. Sci. China Life Sci..

[13] Zhao F., Qi J., Schuster S.C. (2009). Tracking the past: interspersed repeats in an extinct Afrotherian mammal, Mammuthus primigenius. Genome Res..

[14] Jebb D., Huang Z., Pippel M., Hughes G.M., Lavrichenko K., Devanna P., Winkler S., Jermiin L.S., Skirmuntt E.C., Katzourakis A. (2020). Six reference-quality genomes reveal evolution of bat adaptations. Nature.

[15] Liu G.-M., Pan Q., Du J., Zhu P.-F., Liu W.-Q., Li Z.-H., Wang L., Hu C.-Y., Dai Y.-C., Zhang X.-X. (2023). Improved mammalian family phylogeny using gap-rare multiple sequence alignment: A timetree of extant placentals and marsupials. Zool. Res..

[16] Peng C., Ren J.-L., Deng C., Jiang D., Wang J., Qu J., Chang J., Yan C., Jiang K., Murphy R.W. (2020). The genome of Shaw’s sea snake (Hydrophis curtus) reveals secondary adaptation to its marine environment. Mol. Biol. Evol..

[17] Yuan Y., Yang L., Liu T., Zhang H., Lu Q. (2019). Osteoclastogenesis inhibition by mutated IGSF23 results in human osteopetrosis. Cell Prolif..

[18] Unger S., Górna M.W., Le Béchec A., Do Vale-Pereira S., Bedeschi M.F., Geiberger S., Grigelioniene G., Horemuzova E., Lalatta F., Lausch E. (2013). FAM111A mutations result in hypoparathyroidism and impaired skeletal development. Am. J. Hum. Genet..

[19] Castellanos A., Pujol-Coma A., Andres-Bilbe A., Negm A., Callejo G., Soto D., Noël J., Comes N., Gasull X. (2020). TRESK background K^+^ channel deletion selectively uncovers enhanced mechanical and cold sensitivity. J. Physiol..

[20] Chen J., Den Z., Koch P.J. (2008). Loss of desmocollin 3 in mice leads to epidermal blistering. J. Cell Sci..

[21] Favre B., Begre N., Borradori L. (2018). A recessive mutation in the DSP gene linked to cardiomyopathy, skin fragility and hair defects impairs the binding of desmoplakin to epidermal keratins and the muscle-specific intermediate filament desmin. Br. J. Dermatol..

[22] Farasat S., Wei M.-H., Liewehr D.J., Herman M., Steinberg S.M., Bale S., Fleckman P., Toro J. (2009). Novel transglutaminase-1 mutations and genotype-phenotype investigations of 104 patients with autosomal recessive congenital ichthyosis in the USA. J. Med. Genet..

[23] Vandenberg P., Khillan J.S., Prockop D.J., Helminen H., Kontusaari S., Ala-Kokko L. (1991). Expression of a partially deleted gene of human type II procollagen (COL2A1) in transgenic mice produces a chondrodysplasia. Proc. Natl. Acad. Sci. USA.

[24] Ji Q., Li S.-J., Zhao J.-B., Xiong Y., Du X.-H., Wang C.-X., Lu L.-M., Tan J.-Y., Zhu Z.-R. (2023). Genetic and neural mechanisms of sleep disorders in children with autism spectrum disorder: a review. Front. Psychiatry.

[25] Wu T., Deme L., Zhang Z., Huang X., Xu S., Yang G. (2022). Decay of TRPV3 as the genomic trace of epidermal structure changes in the land-to-sea transition of mammals. Ecol. Evol..

[26] Graham A. (2005).

[27] Fischer J. (2009). Autosomal recessive congenital ichthyosis. J. Invest. Dermatol..

[28] Lopes-Marques M., Machado A.M., Alves L.Q., Fonseca M.M., Barbosa S., Sinding M.-H.S., Rasmussen M.H., Iversen M.R., Frost Bertelsen M., Campos P.F. (2019). Complete inactivation of sebum-producing genes parallels the loss of sebaceous glands in Cetacea. Mol. Biol. Evol..

[29] Parsons E.C., Bauer A., McCafferty D., Simmonds M.P., Wright A.J. (2013).

[30] Le Duc D., Velluva A., Cassatt-Johnstone M., Olsen R.-A., Baleka S., Lin C.-C., Lemke J.R., Southon J.R., Burdin A., Wang M.-S. (2022). Genomic basis for skin phenotype and cold adaptation in the extinct Steller’s sea cow. Sci. Adv..

[31] Steinbinder J., Sachslehner A.P., Holthaus K.B., Eckhart L. (2024). Comparative genomics of sirenians reveals evolution of filaggrin and caspase-14 upon adaptation of the epidermis to aquatic life. Sci. Rep..

[32] Bolling M.C., Jan S.Z., Pasmooij A.M., Lemmink H.H., Franke L.H., Yenamandra V.K., Sinke R.J., van den Akker P.C., Jonkman M.F. (2018). Generalized ichthyotic peeling skin syndrome due to FLG2 mutations. J. Invest. Dermatol..

[33] Wohlfart S., Hammersen J., Schneider H. (2016). Mutational spectrum in 101 patients with hypohidrotic ectodermal dysplasia and breakpoint mapping in independent cases of rare genomic rearrangements. J. Hum. Genet..

[34] Hume D.A., Batoon L., Sehgal A., Keshvari S., Irvine K.M. (2022). CSF1R as a Therapeutic Target in Bone Diseases: Obvious but Not so Simple. Curr. Osteoporos. Rep..

[35] Xing W., Liu J., Cheng S., Vogel P., Mohan S., Brommage R. (2013). Targeted disruption of leucine-rich repeat kinase 1 but not leucine-rich repeat kinase 2 in mice causes severe osteopetrosis. J. Bone Miner. Res..

[36] Kendall-Bar J.M., Williams T.M., Mukherji R., Lozano D.A., Pitman J.K., Holser R.R., Keates T., Beltran R.S., Robinson P.W., Crocker D.E. (2023). Brain activity of diving seals reveals short sleep cycles at depth. Science.

[37] Huelsmann M., Hecker N., Springer M.S., Gatesy J., Sharma V., Hiller M. (2019). Genes lost during the transition from land to water in cetaceans highlight genomic changes associated with aquatic adaptations. Sci. Adv..

[38] Sato T.K., Panda S., Miraglia L.J., Reyes T.M., Rudic R.D., McNamara P., Naik K.A., FitzGerald G.A., Kay S.A., Hogenesch J.B. (2004). A functional genomics strategy reveals Rora as a component of the mammalian circadian clock. Neuron.

[39] Etain B., Jamain S., Milhiet V., Lajnef M., Boudebesse C., Dumaine A., Mathieu F., Gombert A., Ledudal K., Gard S. (2014). Association between circadian genes, bipolar disorders and chronotypes. Chronobiol. Int..

[40] Lyamin O.I., Siegel J.M. (2019). Handbook of behavioral neuroscience.

[41] Deutsch C.J., Castelblanco-Martínez D.N., Cleguer C., Groom R., Marsh H. (2022). Ethology and Behavioral Ecology of Sirenia Ethology and Behavioral Ecology of Marine Mammals.

[42] Zeh D.R., Heupel M.R., Hamann M., Jones R., Limpus C.J., Marsh H. (2018). Evidence of behavioural thermoregulation by dugongs at the high latitude limit to their range in eastern Australia. J. Exp. Mar. Biol. Ecol..

[43] Erdsack N., Galves J.A., Powell J.E. (2023). Heat loss or heat uptake? Skin temperature in Antillean manatees (Trichechus manatus manatus, Sirenia: Trichechidae) in Belize. Rev. Biol. Trop..

[44] Deutsch C.J., Castelblanco-Martínez D.N., Groom R., Cleguer C., Marsh H. (2022). Ethology and Behavioral Ecology of Sirenia Ethology and Behavioral Ecology of Marine Mammals.

[45] Tian R., Zhang Y., Kang H., Zhang F., Jin Z., Wang J., Zhang P., Zhou X., Lanyon J., Sneath H. (2023). Sirenian Genomes Illuminate the Evolution of Fully Aquatic Species within the Mammalian Superorder Afrotheria. bioRxiv.

[46] Guilderson T.P., Fairbanks R.G., Rubenstone J.L. (1994). Tropical Temperature Variations Since 20,000 Years Ago: Modulating Interhemispheric Climate Change. Science.

[47] Runge M.C., Sanders-Reed C.A., Langtimm C.A., Hostetler J.A., Martin J., Deutsch C.J., Ward-Geiger L.I., Mahon G.L. (2017).

[48] Baker D.N., Abueg L., Escalona M., Farquharson K.A., Lanyon J.M., Le Duc D., Schöneberg T., Absolon D., Sims Y., Fedrigo O. (2024). A chromosome-level genome assembly for the dugong (Dugong dugon). J. Hered..

[49] Chen S., Zhou Y., Chen Y., Gu J. (2018). fastp: an ultra-fast all-in-one FASTQ preprocessor. Bioinformatics.

[50] Hahn C., Bachmann L., Chevreux B. (2013). Reconstructing mitochondrial genomes directly from genomic next-generation sequencing reads—a baiting and iterative mapping approach. Nucleic Acids Res..

[51] Bernt M., Donath A., Jühling F., Externbrink F., Florentz C., Fritzsch G., Pütz J., Middendorf M., Stadler P.F. (2013). MITOS: improved de novo metazoan mitochondrial genome annotation. Mol. Phylogenet. Evol..

[52] Greiner S., Lehwark P., Bock R. (2019). OrganellarGenomeDRAW (OGDRAW) version 1.3. 1: expanded toolkit for the graphical visualization of organellar genomes. Nucleic Acids Res..

[53] Ranwez V., Douzery E.J., Cambon C., Chantret N., Delsuc F. (2018). MACSE v2: toolkit for the alignment of coding sequences accounting for frameshifts and stop codons. Mol. Biol. Evol..

[54] Stamatakis A. (2014). RAxML version 8: a tool for phylogenetic analysis and post-analysis of large phylogenies. Bioinformatics.

[55] Liu B., Shi Y., Yuan J., Hu X., Zhang H., Li N., Li Z., Chen Y., Mu D., Fan W. (2020). Estimation of genomic characteristics by analyzing k-mer frequency in de novo genome projects. arXiv.

[56] Cheng H., Concepcion G.T., Feng X., Zhang H., Li H. (2021). Haplotype-resolved de novo assembly using phased assembly graphs with hifiasm. Nat. Methods.

[57] Guan D., McCarthy S.A., Wood J., Howe K., Wang Y., Durbin R. (2020). Identifying and removing haplotypic duplication in primary genome assemblies. Bioinformatics.

[58] Zhou C., McCarthy S.A., Durbin R. (2023). YaHS: yet another Hi-C scaffolding tool. Bioinformatics.

[59] Durand N.C., Robinson J.T., Shamim M.S., Machol I., Mesirov J.P., Lander E.S., Aiden E.L. (2016). Juicebox provides a visualization system for Hi-C contact maps with unlimited zoom. Cell Syst..

[60] Benson G. (1999). Tandem repeats finder: a program to analyze DNA sequences. Nucleic Acids Res..

[61] Flynn J.M., Hubley R., Goubert C., Rosen J., Clark A.G., Feschotte C., Smit A.F. (2020). RepeatModeler2 for automated genomic discovery of transposable element families. Proc. Natl. Acad. Sci. USA.

[62] Chen N. (2004). Using RepeatMasker to identify repetitive elements in genomic sequences. Curr Protoc Bioinformatics.

[63] Jurka J., Kapitonov V.V., Pavlicek A., Klonowski P., Kohany O., Walichiewicz J. (2005). Repbase Update, a database of eukaryotic repetitive elements. Cytogenet. Genome Res..

[64] Stanke M., Diekhans M., Baertsch R., Haussler D. (2008). Using native and syntenically mapped cDNA alignments to improve de novo gene finding. Bioinformatics.

[65] Keilwagen J., Hartung F., Grau J., Kollmar M. (2019). Gene Prediction Methods in Molecular Biology.

[66] Haas B.J., Salzberg S.L., Zhu W., Pertea M., Allen J.E., Orvis J., White O., Buell C.R., Wortman J.R. (2008). Automated eukaryotic gene structure annotation using EVidenceModeler and the Program to Assemble Spliced Alignments. Genome Biol..

[67] Buchfink B., Xie C., Huson D.H. (2015). Fast and sensitive protein alignment using DIAMOND. Nat. Methods.

[68] Simão F.A., Waterhouse R.M., Ioannidis P., Kriventseva E.V., Zdobnov E.M. (2015). BUSCO: assessing genome assembly and annotation completeness with single-copy orthologs. Bioinformatics.

[69] Li H., Durbin R. (2009). Fast and accurate short read alignment with Burrows–Wheeler transform. Bioinform..

[70] Li H., Handsaker B., Wysoker A., Fennell T., Ruan J., Homer N., Marth G., Abecasis G., Durbin R., Subgroup, 1000 Genome Project Data Processing (2009). The sequence alignment/map format and SAMtools. Bioinform..

[71] Rhie A., Walenz B.P., Koren S., Phillippy A.M. (2020). Merqury: reference-free quality, completeness, and phasing assessment for genome assemblies. Genome Biol..

[72] Krzywinski M., Schein J., Birol I., Connors J., Gascoyne R., Horsman D., Jones S.J., Marra M.A. (2009). Circos: an information aesthetic for comparative genomics. Genome Res..

[73] Harris R.S. (2007).

[74] Emms D.M., Kelly S. (2019). OrthoFinder: phylogenetic orthology inference for comparative genomics. Genome Biol..

[75] Castresana J. (2000). Selection of conserved blocks from multiple alignments for their use in phylogenetic analysis. Mol. Biol. Evol..

[76] Yang Z. (2007). PAML 4: phylogenetic analysis by maximum likelihood. Mol. Biol. Evol..

[77] Kumar S., Stecher G., Suleski M., Hedges S.B. (2017). TimeTree: a resource for timelines, timetrees, and divergence times. Mol. Biol. Evol..

[78] De Bie T., Cristianini N., Demuth J.P., Hahn M.W. (2006). CAFE: a computational tool for the study of gene family evolution. Bioinformatics.

[79] Xu Z., Wang H. (2007). LTR_FINDER: an efficient tool for the prediction of full-length LTR retrotransposons. Nucleic Acids Res..

[80] Ellinghaus D., Kurtz S., Willhoeft U. (2008). LTRharvest, an efficient and flexible software for de novo detection of LTR retrotransposons. BMC Bioinf..

[81] Ou S., Jiang N. (2018). LTR_retriever: a highly accurate and sensitive program for identification of long terminal repeat retrotransposons. Plant Physiol..

[82] Kirilenko B.M., Munegowda C., Osipova E., Jebb D., Sharma V., Blumer M., Morales A.E., Ahmed A.-W., Kontopoulos D.-G., Hilgers L. (2023). Integrating gene annotation with orthology inference at scale. Science.

[83] Sun Y.-B. (2017). FasParser: a package for manipulating sequence data. Zool. Res..

[84] Zou Z., Zhang J. (2015). Are convergent and parallel amino acid substitutions in protein evolution more prevalent than neutral expectations?. Mol. Biol. Evol..

[85] Kowalczyk A., Meyer W.K., Partha R., Mao W., Clark N.L., Chikina M. (2019). RERconverge: an R package for associating evolutionary rates with convergent traits. Bioinformatics.

[86] Zhou Y., Zhou B., Pache L., Chang M., Khodabakhshi A.H., Tanaseichuk O., Benner C., Chanda S.K. (2019). Metascape provides a biologist-oriented resource for the analysis of systems-level datasets. Nat. Commun..

[87] Hamosh A., Scott A.F., Amberger J.S., Bocchini C.A., McKusick V.A. (2005). Online Mendelian Inheritance in Man (OMIM), a knowledgebase of human genes and genetic disorders. Nucleic Acids Res..

[88] Li H., Durbin R. (2011). Inference of human population history from individual whole-genome sequences. Nature.

[89] Danecek P., Bonfield J.K., Liddle J., Marshall J., Ohan V., Pollard M.O., Whitwham A., Keane T., McCarthy S.A., Davies R.M., Li H. (2021). Twelve years of SAMtools and BCFtools. GigaScience.

[90] McKenna A., Hanna M., Banks E., Sivachenko A., Cibulskis K., Kernytsky A., Garimella K., Altshuler D., Gabriel S., Daly M., DePristo M.A. (2010). The Genome Analysis Toolkit: a MapReduce framework for analyzing next-generation DNA sequencing data. Genome Res..

[91] McGowen M.R., Tsagkogeorga G., Williamson J., Morin P.A., Rossiter J.S. (2020). Positive selection and inactivation in the vision and hearing genes of cetaceans. Mol. Biol. Evol..

[92] Davies K.T., Bennett N.C., Faulkes C.G., Rossiter S.J. (2018). Limited evidence for parallel molecular adaptations associated with the subterranean niche in mammals: a comparative study of three superorders. Mol. Biol. Evol..

[93] Pacifici M., Santini L., Di Marco M., Baisero D., Francucci L., Marasini G.G., Visconti P., Rondinini C. (2013). Generation length for mammals. Nat. Conserv..

[94] Yang L., Wei F., Zhan X., Fan H., Zhao P., Huang G., Chang J., Lei Y., Hu Y. (2022). Evolutionary conservation genomics reveals recent speciation and local adaptation in threatened takins. Mol. Biol. Evol..

