# Supplementary material for: The genome of African manatee Trichechus senegalensis reveals secondary adaptation to the aquatic environment
Source: iScience. 2024 Jun 28;27(7):110394. doi: 10.1016/j.isci.2024.110394 (PMC11292518; doi:10.1016/j.isci.2024.110394)
Supplement: Document S1. Figures S1–S9; Tables S1–S8 and S18–S22 [file mmc1.pdf]

iScience, Volume *27*

Supplemental information

The genome of African manatee *Trichechus senegalensis* reveals secondary adaptation to the aquatic environment

Xin Huang, Guixin Dong, Huizhong Fan, Wenliang Zhou, Guangping Huang, Dengfeng Guan, Delu Zhang, and Fuwen Wei

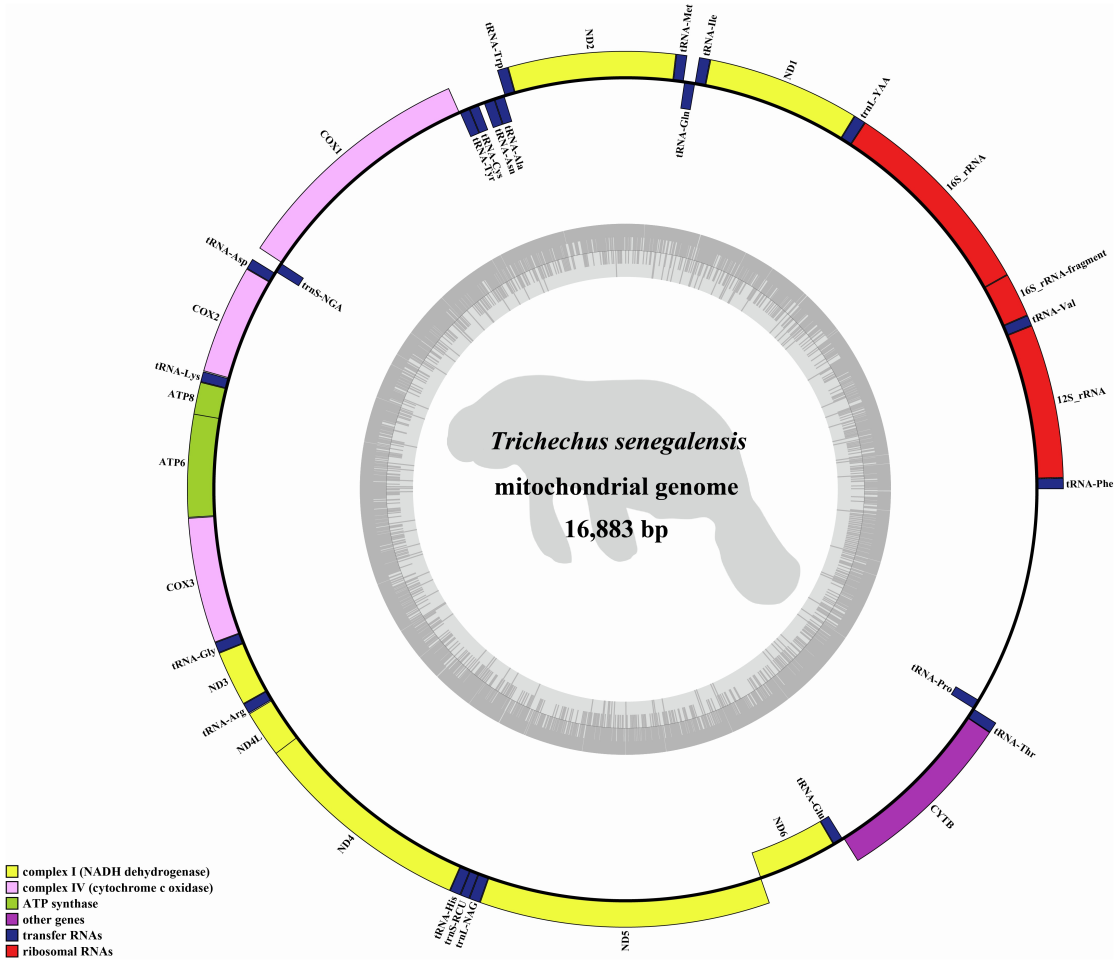

Figure S1. Scheme of the circular mitochondrial genome of the African manatee, related to STAR Methods.

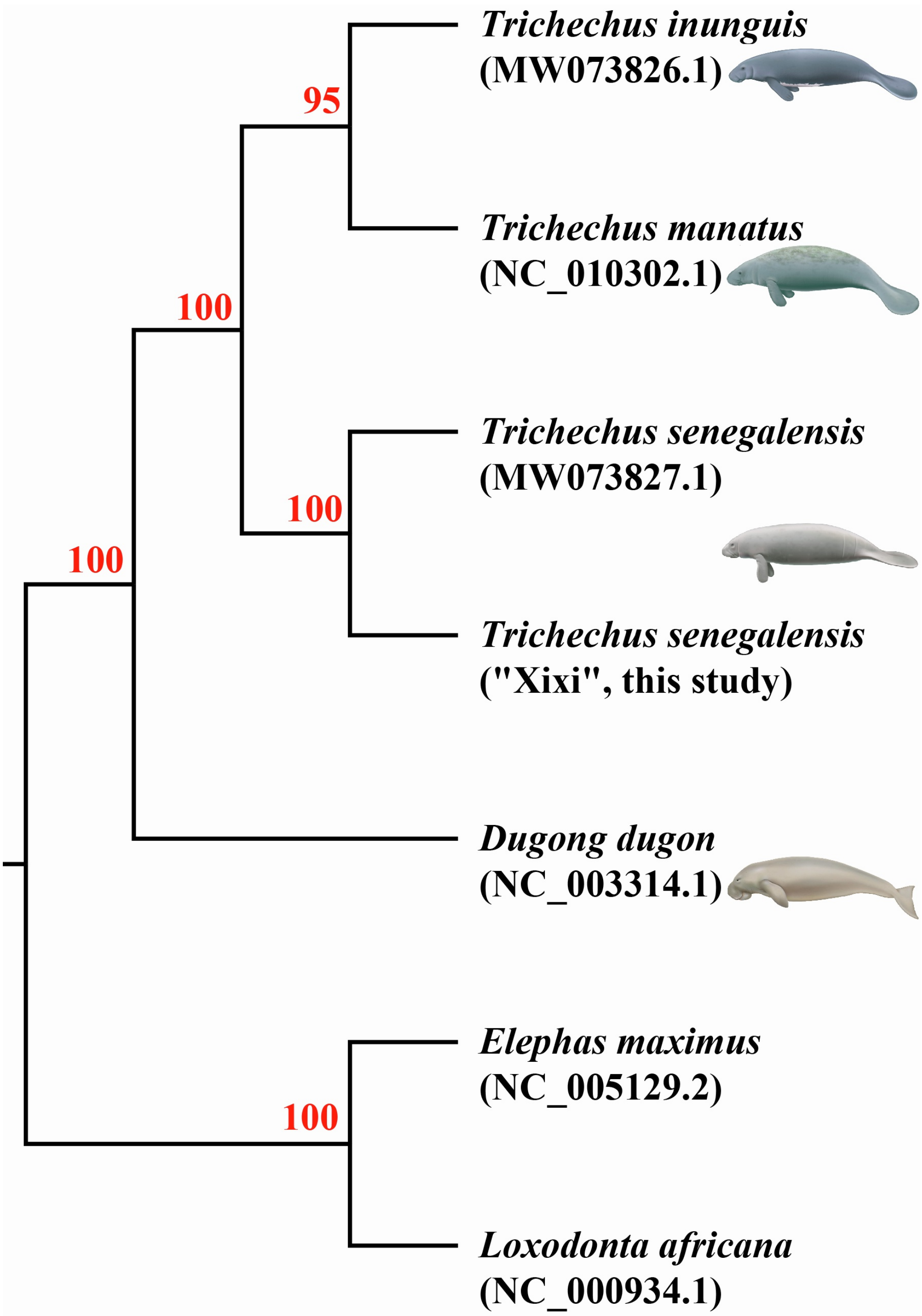

Figure S2. Phylogeny reconstruction of the order Sirenia at the mitochondrial level, related to STAR Methods. The ML tree was reconstructed with RAxML based on the concatenated 13 mitochondrial protein-coding genes. The bootstrap values are shown in red color in each node. The specimen used for genome sequencing was clustered together with the African manatee with high support (bootstrap value = 100).

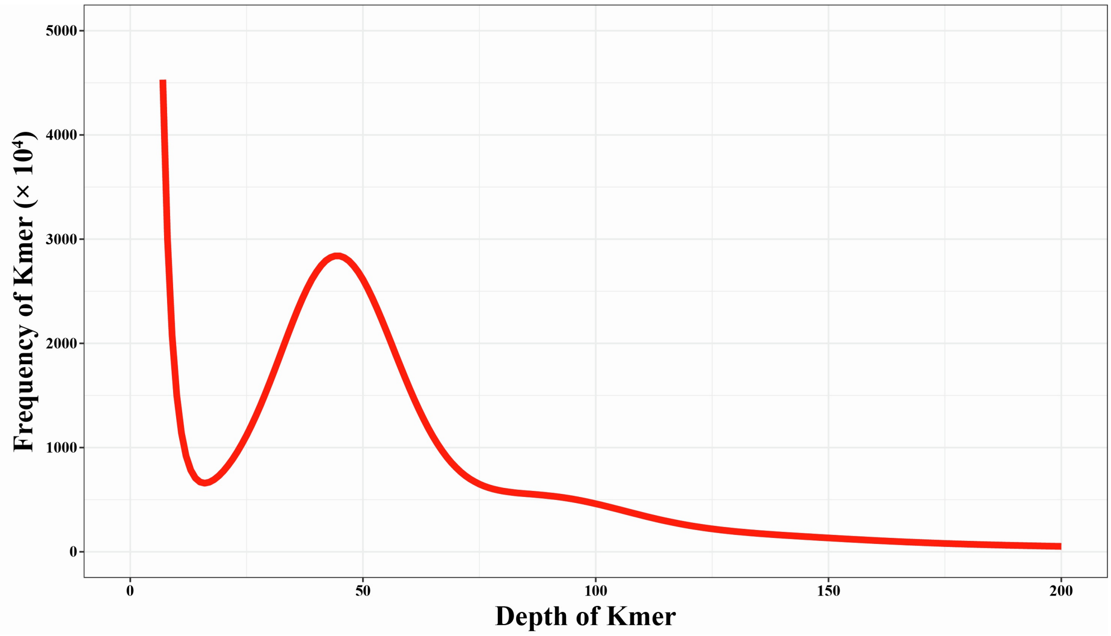

Figure S3. K-mer (k = 17) distribution of the African manatee genome, related to Figure 1.

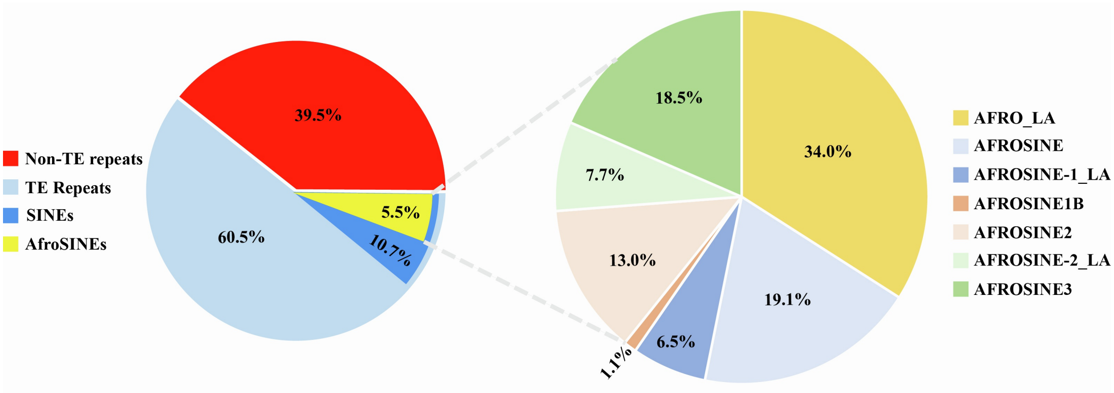

Figure S4. **The percentage of the AfroSINEs identified in the African manatee genome, related to Figure 1.** Different repeated types in AfroSINEs were shown on the right.

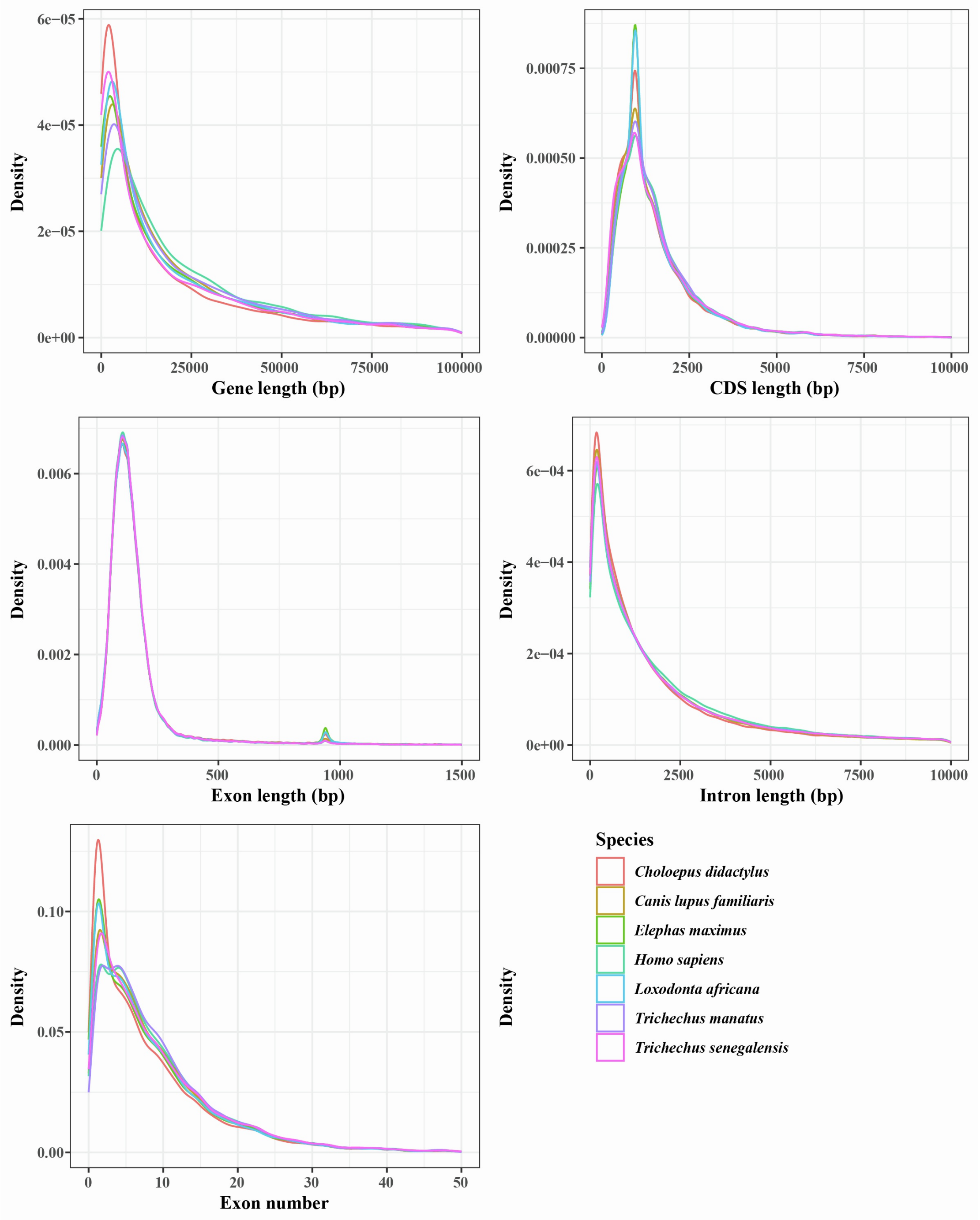

Figure S5. The distribution of the gene length, the CDS length, the exon length, the intron length, and the exon number of each transcript, related to Figure 1.

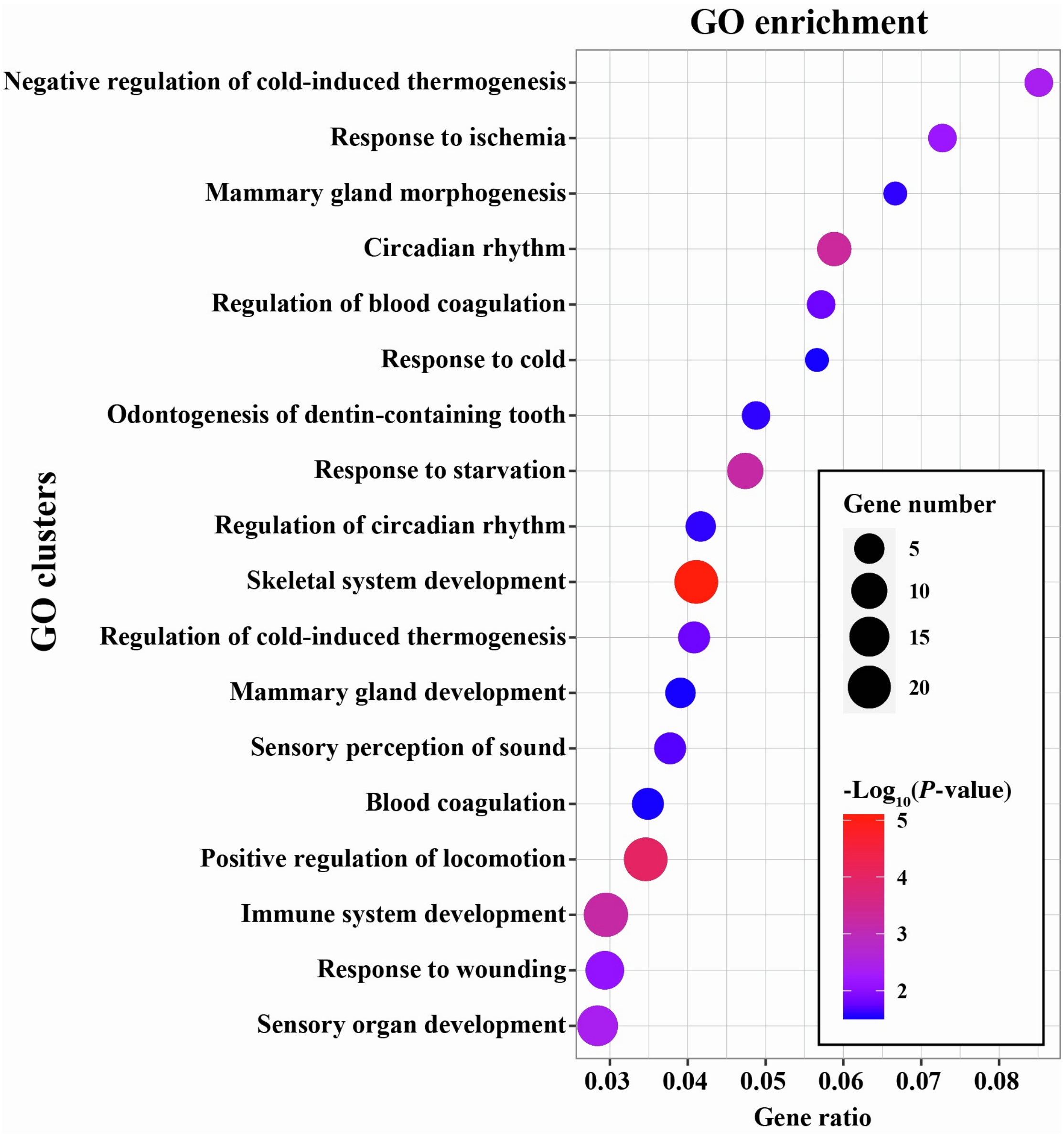

Figure S6. The significantly enriched GO terms for positively selected genes in the African manatee genome, related to Figure 2. Visualization as Figure 2C.

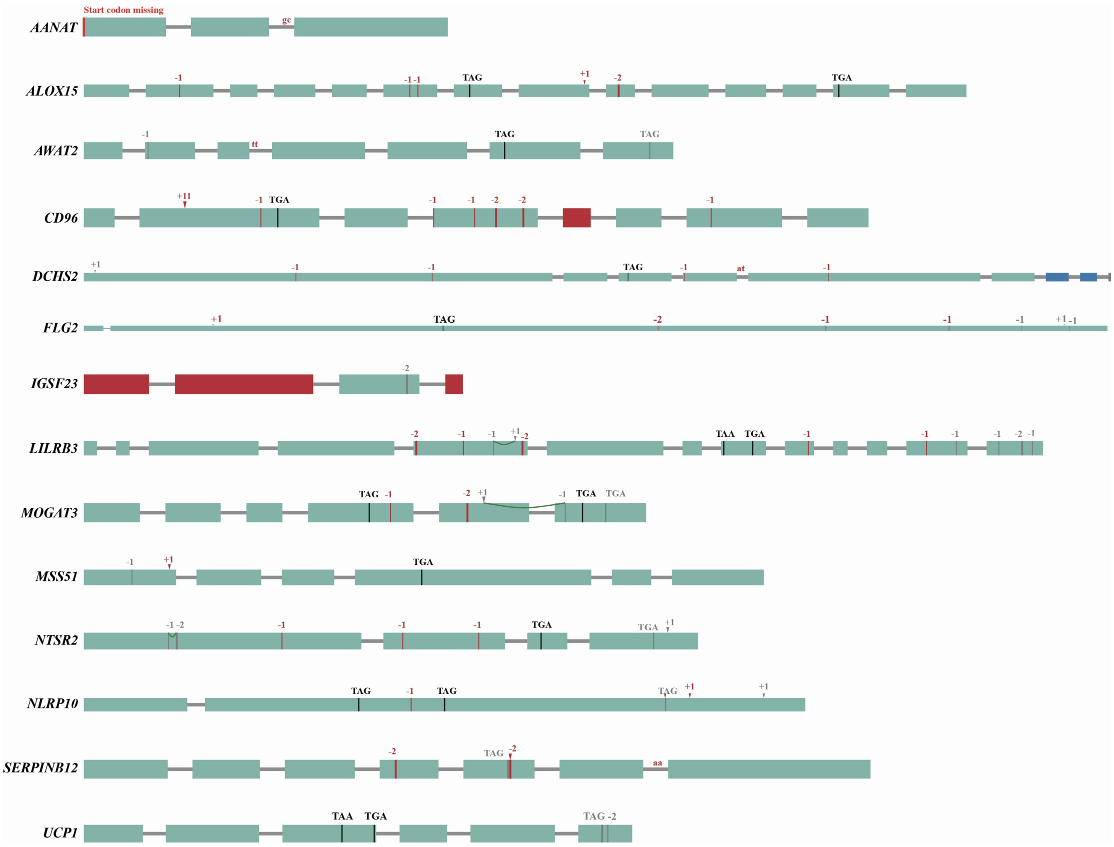

Figure S7. Inactivating mutations in gene *AANAT*, *ALOX15*, *AWAT2*, *CD96*, *DCHS2*, *FLG2*, *IGSF23*, *LILRB3*, *MOGAT3*, *MSS51*, *NTSR2*, *NLRP10*, *SERPINB12*, and *UCP1* of the African manatee, related to Figure 2. Visualization as Figure 2E.

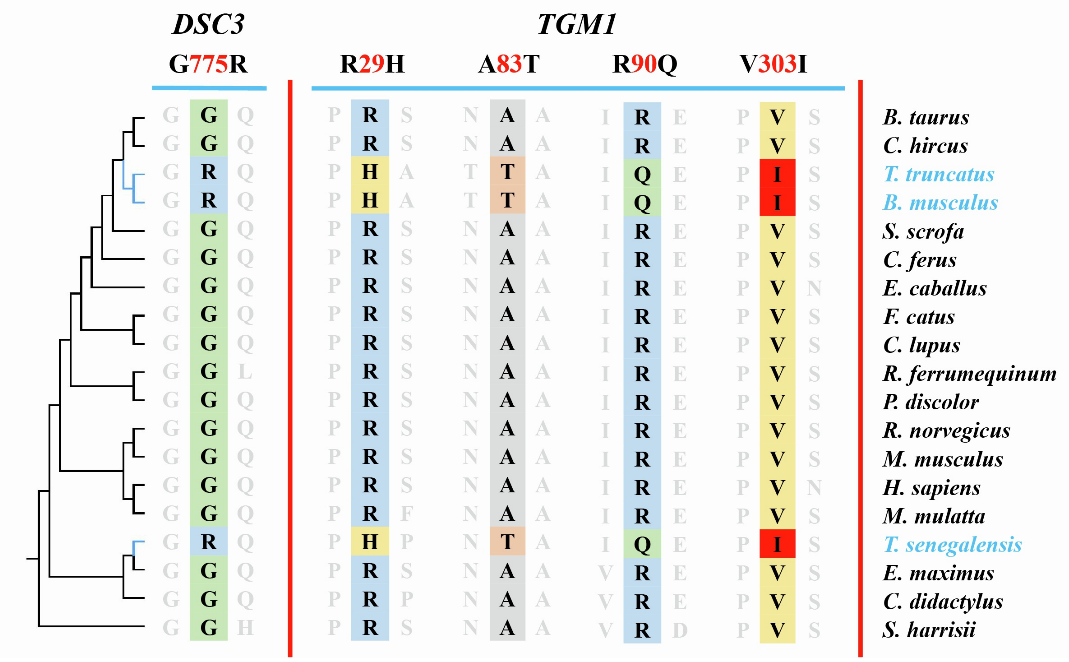

Figure S8. The convergent amino acid substitutions in genes *DSC3* and *TGM1* of fully aquatic mammals, related to Figure 3.

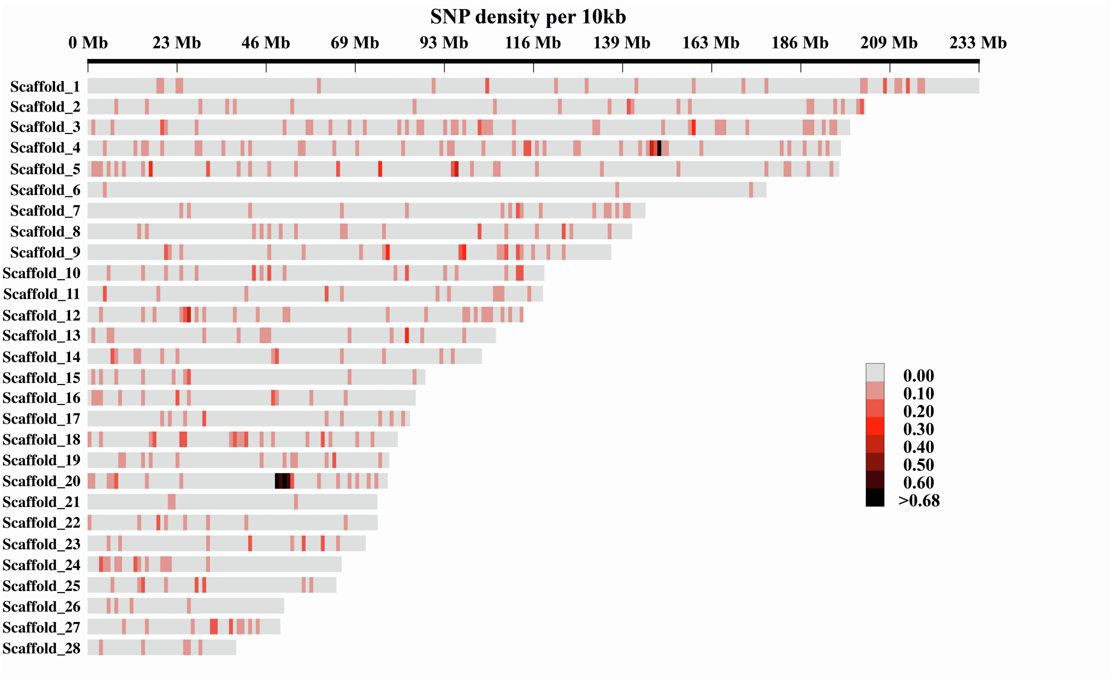

Figure S9. The density of SNPs per 10 kb across chromosome-length scaffolds of the African manatee, related to Figure 4.

Table S1. Summary of whole genome sequenced reads, related to Table 1.

Data type	Cell/Pair	Sequence number	Sum of lengths (bp)	Minimum length (bp)	Average length (bp)	Maximum length (bp)
HiFi	m64181_220520_085259	1,917,306	31,925,492,205	655	16,651.20	67,560
	m64181_220521_194732	1,836,456	30,599,647,460	369	16,662.30	63,080
	m64181_220523_064416	1,583,416	25,898,052,414	573	16,355.80	67,980
	m64446e_220513_084159	1,139,547	19,204,851,524	631	16,853.10	63,775
	m64446e_220514_193602	894,331	14,874,010,224	680	16,631.40	57,381
	m64446e_220517_091820	1,205,217	19,395,897,152	182	16,093.30	61,988
Hi-C	R1	1,231,225,770	184,683,865,500	150	150	150
	R2	1,231,225,770	184,683,865,500	150	150	150
Illumina	R1	543,669,683	81,550,452,450	150	150	150
	R2	543,669,683	81,550,452,450	150	150	150

Table S2. Length of the 28 pseudochromosomes of the African manatee genome, related to Figure 1.

Type	Scaffold name	Length (bp)	Pseudochromosome name
Pseudochromosome	Scaffold_1	233,018,420	Chr1
	Scaffold_2	203,250,200	Chr2
	Scaffold_3	199,232,634	Chr3
	Scaffold_4	196,794,457	Chr4
	Scaffold_5	196,358,007	Chr5
	Scaffold_6	177,308,397	ChrX
	Scaffold_7	145,687,139	Chr6
	Scaffold_8	142,222,000	Chr7
	Scaffold_9	136,758,213	Chr8
	Scaffold_10	119,264,650	Chr9
	Scaffold_11	118,899,908	Chr10
	Scaffold_12	113,759,247	Chr11
	Scaffold_13	106,591,697	Chr12
	Scaffold_14	102,939,642	Chr13
	Scaffold_15	88,142,921	Chr14
	Scaffold_16	85,612,991	Chr15
	Scaffold_17	84,110,478	Chr16
	Scaffold_18	80,919,913	Chr17
	Scaffold_19	78,697,699	Chr18
	Scaffold_20	78,268,128	Chr19
	Scaffold_21	75,604,221	Chr20
	Scaffold_22	75,693,831	Chr21
	Scaffold_23	72,542,000	Chr22
	Scaffold_24	66,255,986	Chr23
	Scaffold_25	64,861,969	Chr24
	Scaffold_26	51,220,874	Chr25
	Scaffold_27	50,201,775	Chr26
	Scaffold_28	38,686,079	Chr27
Unplaced scaffold	Scaffold_29	910,287	unplaced_scaffold_1
	Scaffold_30	647,422	unplaced_scaffold_2
	Scaffold_31	402,282	unplaced_scaffold_3
	Scaffold_32	226,713	unplaced_scaffold_4
	Scaffold_33	158,269	unplaced_scaffold_5
	Scaffold_34	102,165	unplaced_scaffold_6
	Scaffold_35	85,603	unplaced_scaffold_7
	Scaffold_36	64,000	unplaced_scaffold_8
	Scaffold_37	63,279	unplaced_scaffold_9
	Scaffold_38	53,000	unplaced_scaffold_10
	Scaffold_39	36,223	unplaced_scaffold_11

Table S3. Coverage depth for sequenced reads, related to Table 1.

**Coverage depth for HiFi reads**	
**Depth (X)**	**Coverage ratio (%)**
1	100%
4	99.99%
10	99.99%
20	99.81%
Coverage depth (×)	44.5514
Mapping rate (%)	100%

**Coverage depth for NGS reads**	
**Depth (X)**	**Coverage ratio (%)**
1	99.85%
5	99.32%
10	97.87%
20	94.07%
Coverage depth (×)	50.0252
Mapping rate (%)	99.91%

Table S4. Assessment of the African manatee genome completeness using BUSCO, related to Table 1. Completeness evaluation of the assembled genome was based on "mammalia_odb10" dataset in BUSCO software.

Type	Number	Percent (%)
Complete BUSCOs (C)	8,885	96.3
Complete and single-copy BUSCOs (S)	8,801	95.4
Complete and duplicated BUSCOs (D)	84	0.9
Fragmented BUSCOs (F)	81	0.9
Missing BUSCOs (M)	260	2.8
Total BUSCO groups searched	9,226	100

Table S5. Repeat content of the African manatee genome estimated by RepeatMasker software, related to Figure 1.

Type	Super family	Number of elements	Length of sequence (bp)	Percentage of sequence (%)
SINEs		1,998,302	311,262,245	10
	Alu/B1	7	396	0
	MIRs	562,887	76,371,731	2
LINEs		1,678,457	1,028,482,630	32
	LINE1	937,452	715,751,952	22
	LINE2	426,216	103,210,041	3
	L3/CR1	55,305	11,775,216	0
	RTE	258,238	197,510,326	6
LTR elements		628,166	274,344,453	9
	ERVL	148,094	88,340,305	3
	ERVL-MaLRs	324,307	144,095,618	5
	ERV_classI	50,797	25,491,630	1
	ERV_classII	73,722	7,859,498	0
DNA elements		452,822	94,276,130	3
	hAT-Charlie	234,163	94,276,130	1
	TcMar-Tigger	86,691	23,488,891	1
Unclassified		11,415	1,973,071	0
Total interspersed repeats			1,710,338,529	54
Small RNA		48	4,773	0
Satellites		177,862	103,822,642	3
Simple repeats		0	0	0
Low complexity		0	0	0
Total repeats			1,814,613,450	57

Table S6. Repeat sequence statistics of the African manatee genome integrated by four software, related to Figure 1.

Software	Repeat Size (bp)	Percentage of sequence (%)
TRF	48,625,231	1.526
RepeatMasker	1,814,609,246	56.962
RepeatProteinMask	632,041,885	19.84
RepeatModeler	1,731,632,266	54.357
Total	1,965,196,057	61.689

Table S7. Basic statistics of the protein-coding genes for the African manatee and related species, related to Figure 1.

Scientific name	English name	Refseq accession	Total number of genes	Average gene Length (bp)	Average CDS length (bp)	Average CDS number per gene	Average CDS length (bp)	Average intron length (bp)
Trichechus senegalensis	African manatee	This study	20,590	52,066.52	1,678.93	9.69	173.31	5,799.93
Bos taurus	Cattle	GCF_002263795.3	20,976	53,243.84	1,678.34	9.46	177.40	5,001.99
Choloepus didactylus	Linnaeus's two-toed sloth	GCF_015220235.1	23,536	54,557.99	1,593.41	8.54	186.59	5,805.38
Canis lupus familiaris	Dog	GCF_011100685.1	20,950	52,657.04	1,683.14	9.54	176.49	4,795.65
Elephas maximus	Asian elephant	GCF_024166365.1	21,809	60,528.80	1,670.96	9.05	184.63	5,893.54
Homo sapiens	Human	GCF_009914755.1	20,042	61,830.58	1,735.43	9.84	176.38	5,457.10
Loxodonta africana	African savanna elephant	GCF_000001905.1	21,081	51,694.83	1,639.09	9.10	180.13	5,366.00
Trichechus manatus	West Indian manatee	GCF_000243295.1	19,095	57,826.51	1,712.99	9.88	173.43	5,603.70

Table S8. Assessment of the African manatee genome annotation completeness using BUSCO, related to Table 1. Completeness evaluation of the assembled genome annotation was based on "mammalia_odb10" dataset in BUSCO software.

**Type**	**Number**	**Percent (%)**
Complete BUSCOs (C)	9,040	98
Complete and single-copy BUSCOs (S)	8,978	97.3
Complete and duplicated BUSCOs (D)	62	0.7
Fragmented BUSCOs (F)	5	0.1
Missing BUSCOs (M)	181	1.9
Total BUSCO groups searched	9,226	100

Table S18. The IUCN status and genome-wide heterozygosity (π) of three Sirenia species and 25 other mammalian species, related to Figure 4.

Common name	Species name	IUCN status	Observed pi	Source	DOI
Baiji	*Lipotes vexillifer*	CR	0.000121	Zhou et al., 2013	10.1038/ncomms3708
Brown Hyena	*Parahyaena brunnea*	NT	0.000121	Westbury et al., 2018	10.1093/molbev/msy037
Narwhal	*Monodon monoceros*	LC	0.00014	Westbury et al., 2019	10.1016/j.isci.2019.03.023
Iberian lynx	*Lynx pardinus*	EN	0.00018	Westbury et al., 2018	10.1093/molbev/msy037
Cheetah	*Acinonyx jubatus*	VU	0.0002	Dobrynin et al., 2015	10.1186/s13059-015-0837-4
Killer whale	*Orcinus orca*	DD	0.00021	Westbury et al., 2018	10.1093/molbev/msy037
Snow leopard	*Panthera uncia*	VU	0.00023	Cho et al., 2013	10.1038/ncomms3433
Beluga whale	*Delphinapterus leucas*	LC	0.00029	Westbury et al., 2019	10.1016/j.isci.2019.03.023
Tasmanian devil	*Sarcophilus harrisii*	EN	0.00032	Miller et al., 2011	10.1073/pnas.1102838108
Polar bear	*Ursus maritimus*	VU	0.00032	Westbury et al., 2018	10.1093/molbev/msy037
Amur tiger	*Panthera tigris altaica*	EN	0.000486	Cho et al., 2013	10.1038/ncomms3433
African manatee	*Trichechus senegalensis*	VU	0.000497	This study	This study
African lion	*Panthera leo*	VU	0.000583	Cho et al., 2013	10.1038/ncomms3433
Minke whale	*Balaenoptera acutorostrata*	LC	0.00061	Yim et al., 2014	10.1038/ng.2835
Eastern lowland gorilla	*Gorilla beringei graueri*	CR	0.00064	Xue et al., 2015	10.1126/science.aaa3952
Chinese pangolin	*Manis pentadactyla*	CR	0.00085	Heighton et al., 2023	10.1093/molbev/msad190
Indo-Pacific finless porpoise	*Neophocaena phocaenoides*	VU	0.00093	Morin et al., 2020	10.1111/1755-0998.13284
Indo-Pacific bottlenose dolphin	*Tursiops aduncus*	NT	0.00095	Vijay et al., 2018	10.1093/molbev/msy108

Yangtze finless porpoise	*Neophocaena asiaeorientalis asiaeorientalis*	EN	0.00105	Morin et al., 2020	10.1111/1755-0998.13284
Florida manatee	*Trichechus manatus latirostris*	VU	0.00106	This study	This study
Giant panda	*Ailuropoda melanoleuca*	VU	0.00135	Leffler et al., 2012	10.1371/journal.pbio.1001388
Bottlenose dolphin	*Tursiops truncatus*	LC	0.00142	Yim et al., 2014	10.1038/ng.2835
Fin whale	*Balaenoptera physalus*	VU	0.00151	Yim et al., 2014	10.1038/ng.2835
Dugong	*Dugong dugon*	VU	0.00166	This study	This study
Olive baboon	*Papio anubis*	LC	0.00189	Corbett-Detig_2015	10.1371/journal.pbio.1002112
Blue whale	*Balaenoptera musculus*	EN	0.0021	Morin et al., 2020	10.1111/1755-0998.13284
Sperm whale	*Physeter catodon*	VU	0.00228	Morin et al., 2020	10.1111/1755-0998.13284
Przewalski’s horse	*Equus ferus przewalskii*	EN	0.00363	Corbett-Detig et al., 2015	10.1371/journal.pbio.1002112

Table S19. The mitochondrial genome accession for phylogeny reconstruction of the order Sirenia, related to STAR Methods.

Scientific name	English name	Accession number
Trichechus senegalensis	African manatee	MW073827.1
Trichechus senegalensis	African manatee	This study
Trichechus manatus	West Indian manatee	NC_010302.1
Trichechus inunguis	Amazonian manatee	MW073826.1
Dugong dugon	Dugong	NC_003314.1
Loxodonta africana	African elephant	NC_000934.1
Elephas maximus	Asian elephant	NC_005129.2

Table S20. Genome accession for the mammals used in genome-wide comparative genomic analysis, related to STAR Methods.

Order	Scientific name	English name	Abbr.	GenBank assembly accession	RefSeq assembly accession
Artiodactyla	*Balaenoptera musculus*	Blue whale	Bmus	GCA_009873245.2	GCF_009873245.2
Artiodactyla	*Bos taurus*	Cattle	Btau	GCA_002263795.4	GCF_002263795.3
Artiodactyla	*Camelus ferus*	Wild Bactrian camel	Cfer	GCA_009834535.1	GCF_009834535.1
Artiodactyla	*Capra hircus*	Goat	Chir	GCA_001704415.2	GCF_001704415.2
Artiodactyla	*Sus scrofa*	Wild boar	Sscr	GCA_000003025.6	GCF_000003025.6
Artiodactyla	*Tursiops truncatus*	Common bottlenose dolphin	Ttru	GCA_011762595.1	GCF_011762595.1
Carnivora	*Canis lupus familiaris*	Dog	Clup	GCA_014441545.1	GCF_014441545.1
Carnivora	*Felis catus*	Cat	Fcat	GCA_018350175.1	GCF_018350175.1
Chiroptera	*Phyllostomus discolor*	Pale spear-nosed bat	Pdis	GCA_004126475.2	GCF_004126475.2
Chiroptera	*Rhinolophus ferrumequinum*	Greater horseshoe bat	Rfer	GCA_004115265.2	GCF_004115265.2
Dasyuromorphia	*Sarcophilus harrisii*	Tasmanian devil	Shar	GCA_902635505.1	GCF_902635505.1
Perissodactyla	*Equus caballus*	Horse	Ecab	GCA_002863925.1	GCF_002863925.1
Pilosa	*Choloepus didactylus*	Linnaeus's two-toed sloth	Cdid	GCA_015220235.1	GCF_015220235.1
Primates	*Homo sapiens*	Human	Hsap	GCA_009914755.1	GCF_009914755.1
Primates	*Macaca mulatta*	Rhesus macaque	Mmul	GCA_003339765.1	GCF_003339765.1
Proboscidea	*Elephas maximus*	Asian elephant	Emax	GCA_024166365.1	GCF_024166365.1
Rodentia	*Rattus norvegicus*	Brown rat	Rnor	GCA_015227675.2	GCF_015227675.2
Rodentia	*Mus musculus*	House mouse	Mmus	GCA_000001635.27	GCF_000001635.27
Sirenia	*Trichechus senegalensis*	African manatee	Tsen	This study	This study

Table S21. Fossil-calibrated time points for calculating divergence time when reconstructing the phylogeny of the class Mammalia, related to STAR Methods.

Node	Node minimum age (Ma)	Node maximum age (Ma)
Felis catus - *Canis lupus familiaris* split	52.9	57.3
Balaenoptera musculus - *Tursiops truncatus* split	32.3	35.2
Bos taurus - *Capra hircus* split	19.5	30.9
Phyllostomus discolor - *Rhinolophus ferrumequinum* split	57.9	64
Rattus norvegicus - *Mus musculus split*	14	19
Homo sapiens - *Macaca mulatta* split	27	30.6
Elephas maximus - *Trichechus senegalensis* split	56.9	64.2
Homo sapiens - *Sarcophilus harrisii* split	147.1	164.3

Table S22. The reference genome and SRA accession number for PSMC analysis, related to STAR Methods.

Scientific name	GenBank assembly accession	SRA acccesion number	Layout	Platform
Trichechus manatus latirostris	GCA_000243295.1	SRR307134<br>SRR307135<br>SRR307136<br>SRR307137<br>SRR314670<br>SRR328413<br>SRR328414<br>SRR328415<br>SRR328416<br>SRR328417<br>SRR331132<br>SRR331133<br>SRR331135<br>SRR331146<br>SRR331136<br>SRR331138<br>SRR331140<br>SRR331141<br>SRR331143<br>SRR331144<br>SRR331145<br>SRR331147<br>SRR331148	PAIRED	Illumina HiSeq 2000
Dugong dugon	GCA_905400935.1	SRR17854495<br>SRR17870680	PAIRED	Illumina NovaSeq 6000
Trichechus senegalensis	This study	This study	PAIRED	DNBSEQ-T7
